# Construction of embedded fMRI resting-state functional connectivity networks using manifold learning

**DOI:** 10.1007/s11571-020-09645-y

**Published:** 2020-11-03

**Authors:** Ioannis K. Gallos, Evangelos Galaris, Constantinos I. Siettos

**Affiliations:** 1 School of Applied Mathematical and Physical Sciences, National Technical University of Athens, Athens, Greece; 2 Dipartimento di Matematica e Applicazioni “Renato Caccioppoli”, Università degli Studi di Napoli Federico II, Napoli, Italy

**Keywords:** Resting-state fMRI, Functional connectivity networks, Schizophrenia, Manifold learning, Machine learning

## Abstract

We construct embedded functional connectivity networks (FCN) from benchmark resting-state functional magnetic resonance imaging (rsfMRI) data acquired from patients with schizophrenia and healthy controls based on linear and nonlinear manifold learning algorithms, namely, Multidimensional Scaling, Isometric Feature Mapping, Diffusion Maps, Locally Linear Embedding and kernel PCA. Furthermore, based on key global graph-theoretic properties of the embedded FCN, we compare their classification potential using machine learning. We also assess the performance of two metrics that are widely used for the construction of FCN from fMRI, namely the Euclidean distance and the cross correlation metric. We show that diffusion maps with the cross correlation metric outperform the other combinations.

## Introduction

Over the past years, functional magnetic resonance imaging (fMRI) has been widely used for the identification of brain regions that are related to both functional segregation and integration. Regarding functional segregation, the conventional analysis relies on the identification of the activated voxels based on functional response models and multivariate statistics between experimental conditions (e.g. resting-state vs. task-stimulated activity). A representative example is the General Linear Model (GLM) that is implemented in well established software packages such as SPM (Friston et al. 1994) and FSL (Smith et al. 2004). On the other hand, for the assessment of functional integration, there is a distinction between functional and effective connectivity (Friston 2011). Functional connectivity (FC) analysis looks for statistical dependencies (e.g. correlations, coherence) between brain regions. Effective connectivity (EC) analysis tries to reveal the influence that one neural system exerts on another. A detailed review on the differences between FC and EC approaches can be found in Friston (2011).

Here, we focus on the construction of functional connectivity networks (FCN) based on resting-state fMRI (rsfMRI) recordings. In rsfMRI, there is no stimuli and thus the assessment of functional integration is more complex and not so straightforward compared to task-related experiments (Khosla et al. 2019). Furthermore, spontaneous/ resting-state brain activity as measured with fMRI has been considered as a potential biomarker in psychiatric disorders (see e.g. the review of Zhou et al. 2010). In general, two basic frameworks are explored for the construction of FCN: (a) seed-based analysis (SBA) and (b) independent component-based analysis (ICA). In the SBA (Cole et al. 2010), the (averaged) fMRI signals of the regions of interest (ROIs) are correlated with each other; correlations above a threshold are considered functional connections between seeds/ROIs. Even though the SBA has been proved extremely useful in identifying functional networks of specific brain regions (Greicius et al. 2003; Fox et al. 2005; Margulies et al. 2007), its major disadvantage is the requirement of the a-priori knowledge of the functional organization of the brain, while possible correlations between seeds can be due to structured spatial confounds (e.g. scanner artifacts) (Cole et al. 2010). Furthermore, the definition of a seed is based on standard coordinates, while at the subject level, anatomical differences may lead to the consideration of functionally irrelevant voxels at the group level. Despite the use of normalization techniques, the accuracy of this approach is limited especially for brain regions, such as the hippocampus, where neurogenesis continues even in the adult life (Saxe et al. 2006). On the other hand, ICA (Hyvärinen and Oja 2000) has arisen as an alternative approach since the early 2000s (Beckmann et al. 2005; Beckmann and Smith 2005; Kim et al. 2010). ICA decomposes the 4D fMRI data to a set of spatial components with maximum statistical independence and their associated time series. Smith et al. (2009) in a meta-analytic study of 30,000 rsfMRI scans with the aid of ICA revealed a functional “partition” of the brain into resting-state networks (RSNs), such as the sensorimotor, default mode and auditory networks. Applications of ICA include also data pre-processing, where noise-related components are regressed out from the original fMRI signals (Pruim et al. 2015). However, while ICA produces spatial components that are statistically independent to each other, there is no clear link between the spatial components and specific brain functions and spatial components cannot in general be ordered by relative importance (Cole et al. 2010). Another issue is that most of the standard algorithms that compute independent components (ICs) utilize gradient based optimization algorithms that use an iterative scheme; the initial guesses in these algorithms are generated randomly making the whole process stochastic. As a consequence, the obtained spatial components may differ significantly for the same dataset over repeated runs (Himberg et al. 2004). Hence, the reproducibility of the ICA results over repeated runs may be questioned.

In order to tackle the above issues, several techniques have been proposed for the classification of ICs and the construction of subject-specific ROIs (Pamplona et al. 2020; Yang et al. 2008). Advances have also been made regarding the selection of the model order of the ICA decomposition, such as the Bayesian dimensionality estimation technique (Beckmann et al. 2005) and the use of theoretic information criteria for model order selection (Li et al. 2007). Finally, the so-called ranking and averaging ICA by reproducibility (RAICAR) (Yang et al. 2008; Himberg et al. 2004) (see also Cole et al. (2010) for a critical discussion) aims at resolving issues regarding stochasticity and robustness of the ICA decomposition. RAICAR utilizes a sufficient number of ICA realizations and based on the reproducibility of the ICs aims to rank them in terms of the most “reliable” components. Reliable ICs among realizations are assessed via correlations and the final estimate of each component is averaged.

Alternatively and/or complementary to the above analysis, linear manifold learning algorithms such as Principal Component Analysis (PCA) (Jollife 2002; Worsley et al. 2005; Baumgartner et al. 2000) and classical Multidimensional Scaling (MDS) (Kruskal 1964; Friston et al. 1996) have been also exploited. PCA has been succesfully applied in the pre-processing routine for dimensionality reduction (often prior to ICA) (Iraji et al. 2016). Applications of PCA include also the recovery of signals of interest (Viviani et al. 2005) and the construction of FCN from fMRI scans in task-related experiments (Worsley et al. 2005; Baumgartner et al. 2000). In these studies, the performance of PCA with respect to the detection of regions of correlated voxels has been shown to be satisfactory but not without problems. For example, a study by Baumgartner et al. (2000) highlighted the limits of PCA to correctly identify activation of brain regions in cases of low contrast-to-noise ratios (CNR) appearing when signal sources of e.g. physiological noise are present.

MDS has been also widely used in fMRI (mostly for task-based studies) mainly for the identification of similarities between brain regions in terms of voxel-wise connectivity (Shinkareva et al. 2012, 2013; Tzagarakis et al. 2009; O’Toole et al. 2007; Haxby et al. 2001; de Beeck et al. 2010). The implementation of MDS in neuroimaging dates back to the work of Friston et al. (1996), where embedded (voxel-wise) connectivity from PET data was investigated during word generation tasks between healthy and schizophrenia subjects. Salvador et al. (2005) used MDS to investigate the embedded connectivity of anatomical regions of the brain from rsfMRI data. Benjaminsson et al. (2010) used MDS to embed high-dimensional rsfMRI data from the mutual information space to a low dimensional Euclidean space for the identification of RSNs. Hervé et al. (2012) used MDS to acquire a low dimensional approximation of interregional correlations for the investigation of the affective speech comprehension. Finally, in a meta-analytic study by Etkin and Wager (2007), MDS was exploited to provide a low-dimensional visualization of co-activation interrelations of Regions of Interest (ROIs). MDS has been also used in studies investigating the functional (dys)connectivity associated with schizophrenia (Welchew et al. 2002) and Asperger’s Syndrome (Welchew et al. 2005).

However, thus far, only a few studies have exploited non-linear manifold learning algorithms such as Locally Linear Embedding (LLE) (Roweis and Saul 2000), Isometric Feature Mapping (ISOMAP) (Tenenbaum et al. 2000), diffusion maps (Coifman and Lafon 2006) and kernel PCA (kPCA) (Schölkopf et al. 1997) for the analysis of fMRI data and particularly for the construction of FCN. The LLE method has been applied in rsfMRI studies for the improvement of predictions in ageing (Qiu et al. 2015), for the classification of healthy subjects and patients with schizophrenia (Shen et al. 2010) and as an alternative method for dimensionality reduction before the application of ICA in task-related fMRI, where non-linear relationships in the BOLD signal are introduced (Mannfolk et al. 2010). The kPCA method has been recently applied to a fMRI study for non-linear feature extraction (Tsatsishvili et al. 2018). In this study, it was shown that certain important features could not be found by the standard PCA. kPCA has been also used for feature extraction towards the automated diagnosis of (Attention-Deficit Hyperactivity Disorder) ADHD (Sidhu et al. 2012). In Anderson and Cohen (2013), ISOMAP was employed to a benchmark rsfMRI dataset of 146 subjects for the construction of embedded low-dimensional FCN for the classification of controls and schizophrenic subjects. ROIs were selected using single-subject ICA and the similarities between the ICs were assessed using a pseudo-distance measure based on cross correlation. Graph-theoretic measures were then used for the discrimination between patients and healthy controls. Another study based on single-subject ICA exploited ISOMAP to classify spatially unaligned fMRI scans (Anderson et al. 2010). The study focused on comparisons between patients with schizophrenia versus healthy controls and different age groups of healthy controls versus patients with alzheimer’s disease. Despite the relatively low sample sizes, results were promising with good classification rates. Recently, Haak et al. (2018) utilized ISOMAP for the construction of individualised connectopies from rsfMRI recordings taken from the WU-Minn Human Connectome Project in a fully data-driven manner. Only a handful of studies have used diffusion maps for the analysis of fMRI data. These studies have been focused mainly on the clustering of spatial maps of task-related experiments (Shen and Meyer 2005; Sipola et al. 2013). Shen and Meyer (2005), and Sipola et al. (2013) used diffusion maps with a Gaussian kernel to cluster selected fMRI spatial maps that are derived by ICA. The approach was demonstrated using fMRI recordings acquired from healthy participants listening to a stimulus with a rich musical structure. Other applications of diffusion maps in neuroimaging include predicitions of epileptic seizures and the identification of the pre-seizure state in EEG timeseries (Lian et al. 2015; Duncan et al. 2013). A review on the intersection between manifold learning methods and the construction of FCN can be found in Siettos and Starke (2016), and Richiardi et al. (2013).

Here, we employed MDS, ISOMAP, diffusion maps, kPCA and LLE to construct embedded FCN from rsfMRI data taken from healthy controls and schizophrenia patients. For our demonstrations, we used the Center for Biomedical Research Excellence (COBRE) rsfMRI dataset that is publicly available and has been used recently in many studies (Calhoun et al. 2012; Mayer et al. 2013; Anderson and Cohen 2013; Qureshi et al. 2017). Based on key global graph-theoretic measures of the embedded graphs, we assessed their classification efficiency using several machine learning algorithms, namely linear standard Support vector machines (LSVM), radial (radial basis function kernel) support vector machines (RSVM), k-nearest neighbours (k-NN) classifier, and artificial neural networks (ANN). We also investigated their performance considering two commonly used distance metrics, namely the cross correlation and the Euclidean distance. Our analysis showed that diffusion maps with the cross correlation outperformed all other combinations.

At this point, we should note, that our study does not aim at extracting the best classification performance by trying to find the best possible pre-processing pipe-line of the raw fMRI data and/or the selection of “best” subjects and/or the selection of the best set of graph-theoretic measures that provide the maximum classification. Yet, we aim at using state-of-the-art manifold learning methods for the construction of embedded FCN and compare their classification efficiency using only the three fundamental global graph measures, i.e. the average path length, the global clustering coefficient and the degree. Furthermore, our results can be compared to those obtained by similar studies (see e.g. Anderson and Cohen 2013) using the same pipe-line for data pre-processing and single-subject ICA. To the best of our knowledge, this paper is the first to perform such a thorough comparative analysis of both linear and nonlinear manifold learning on rsfMRI data. It is also the first study to show how diffusion maps can be used for the construction of FCN from rsfMRI, assessing also the efficiency of two basic distance metrics, the cross correlation and the Euclidean distance.

## Materials and methods

### Data description

For our demonstrations we used the Schizophrenia COBRE dataset (http://fcon_1000.projects.nitrc.org/indi/retro/cobre.html) comprised of rsfMRI data from 74 healthy and 72 Schizophrenic subjects of varying ages (18–65 years in both groups). All subjects were screened and excluded if they had history of neurological disorders, mental retardation, severe head trauma with more than 5 min loss of consciousness, substance abuse or dependence within the last 12 months. Diagnostic information was collected using the Structured Clinical Interview used for DSM Disorders (SCID).

For the anatomical imaging, a multi-echo Magnetization Prepared RApid Gradient Echo (MPRAGE) sequence was used with the following set of parameters: TR (repetition time)/TE (echo time)/TI (inversion time) = 2530/[1.64, 3.5, 5.36, 7.22, 9.08]/900 ms, flip angle = 7$^{\circ }$, Field Of View (FOV) = $256\times 256$ mm^2^, Slab thickness = 176 mm, data matrix = $256\times 256\times 176$, Voxel size = $1\times 1\times 1$ mm^3^, Number of echos=5, Pixel bandwidth=650 Hz, Total scan time = 6 min. With 5 echoes, the TR, TI and time to encode partitions for the multi-echo MPRAGE are similar to that of a conventional MPRAGE, resulting in similar Gray Matter (GM)/ White Matter (WM)/ CelebroSpinal Fluid (CSF) contrast. The rsfMRI data-set was collected with single-shot full k-space Echo-Planar Imaging (EPI) with ramp sampling correction using the intercomissural line (AC-PC) as a reference (TR: 2 s, TE: 29 ms, slice size: 64x64, number of slices: 32, voxel size: $3\times 3\times 4$ mm$^3$).

### Pre-processing and signal extraction

As also implemented in other studies (see e.g. Anderson and Cohen 2013), we first performed a basic pre-processing of the raw fMRI data using FSL (FMRIB’s Software Library, www.fmrib.ox.ac.uk/fsl). In particular, the following pre-processing steps were applied: motion correction using Fsl’s linear registration tool (MCFLIRT) (Jenkinson et al. 2002), slice-timing correction using Fourier-space time-series phase-shifting; non-brain removal using the brain extraction tool (BET) (Smith 2002), spatial smoothing using a 5 mm full-width at half-maximum (FWHM) Gaussian kernel, grand-mean intensity normalization of the entire 4D dataset by a single multiplicative factor (10,000 divided by the grand mean intensity, Fsl’s default). Furthermore, we used ICA automatic removal of motion artifacts (AROMA) (Pruim et al. 2015) to detect and factor out noise-related (motion artifacts and other structured noise components like cardiac pulsation confounds) ICs. After the implementation of ICA AROMA, we applied a high-pass temporal filtering at 0.01 Hz (100 s) as it is highly recommended (Pruim et al. 2015).

We then proceeded with the decomposition of the pre-processed fMRI data to spatial ICs (for each subject) using the RAICAR methodology (Yang et al. 2008). In this way, we computed the most reproducible spatial ICs over repeated runs as a solution to the well known problem of the variability of the ICA decomposition (Himberg et al. 2004). This choice is related to the benchmark fMRI data per se as there is only a single session per subject with relatively small duration (6 min); therefore we wouldn’t expect a robust ICA decomposition for all subjects (see also the discussion in Cole et al. 2010). Another choice would be to perform group-ICA analysis [which is subject to other limitations (see in the “Discussion” section)], but we decided to use single-subject ICA in order to have a common ground with the methodologically similar work presented in Anderson and Cohen (2013).

### Ranking and averaging ICA by reproducibility (RAICAR)

#### Independent component analysis (ICA)

ICA is a linear data-driven technique that reduces the high-dimensional fMRI *F*(*t*, *x*, *y*, *z*) space in a set of *M* statistically independent components. This reduction can be represented as:

1 $$\begin{aligned} F(t,x,y,z)=\sum _{i=1}^{M} A_{i}(t) C_{i}(x,y,z), \end{aligned}$$

where *F*(*t*, *x*, *y*, *z*) is the measured BOLD signal, ${A_{i}(t)}$ is the temporal amplitude (the matrix ${\mathbf {A}}$ containing all temporal amplitudes is known as mixing matrix) and ${C_{i}(x,y,z)}$ is the spatial magnitude of the i-th ICA component. While PCA requires that the principal components are uncorrelated and orthogonal, ICA asks for statistical independence between the ICs. Generally, ICA algorithms are based either on the minimization of mutual information or the maximization of non-Gaussianity among components. As discussed in the introduction, most of the standard implementations of ICA, such as the one in MELODIC (Multivariate Exploratory Linear Optimized Decomposition into Independent Components) (Beckmann and Smith 2004), which is part of FSL (fMRIB’s Software Library) share similar gradient-based optimization algorithms using an iterative scheme whose initial values are generated randomly, thus making the whole process stochastic. As a consequence, results over repeated runs may differ significantly (Himberg et al. 2004). A solution to this problem is provided by the so-called Ranking and Averaging ICA by Reproducibility (RAICAR) (Yang et al. 2008) that we briefly describe in the following section.

#### Ranking and averaging ICA by reproducibility (RAICAR)

The RAICAR methodology developed by Yang et al. (2008) was introduced to tackle the problem of the ICs variability by performing *K* ICA realizations. Thus, RAICAR leads to *K* “slightly” different mixing matrices ${\mathbf {A}}_{1},{\mathbf {A}}_{2} \dots {\mathbf {A}}_{K}$ and *K* different sets of spatial maps ${\mathbf {S}}_{1},{\mathbf {S}}_{2} \dots {\mathbf {S}}_{K}$. Each realization finds a fixed number *M* of spatial ICs. Then, a cross realization correlation matrix ($\mathbf {CRCM}$) of size $M\cdot K{\times }M\cdot K$ is constructed and the alignment (ICA produces unaligned components) of ICs across realizations takes place on the basis of the absolute maximum spatial correlation among components. Thus, the cross realization correlation matrix reads:

$$\begin{aligned} {\mathbf {CRCM}} = \left[ \begin{array}{ccccc} {\mathbf {R}}_{1,1} &{} {\mathbf {R}}_{1,2} &{} \dots &{} {\mathbf {R}}_{1,K-1} &{} {\mathbf {R}}_{1,K} \\ {\mathbf {R}}_{2,1} &{} &{} \dots &{} \dots &{} {\mathbf {R}}_{2,K} \\ \vdots &{} \dots &{} \ddots &{} \dots &{} \vdots \\ {\mathbf {R}}_{K-1,1} &{} \dots &{} \dots &{} &{} {\mathbf {R}}_{K-1,K} \\ {\mathbf {R}}_{K,1} &{} {\mathbf {R}}_{K,2} &{} \dots &{} {\mathbf {R}}_{K,K-1} &{} {\mathbf {R}}_{K,K} \end{array}\right] \end{aligned}$$

${\mathbf {R}}_{i,j}$ with $i,j=1,2\ldots K$ are submatrices of size $M{\times }M$ and their elements represent the absolute spatial correlation coefficients among components and across realizations. CRCM is a symmetric matrix and its diagonal consists of identity matrices which are ignored for the next steps of the algorithm.

The procedure starts with the identification of the global maximum of the CRCM, thus finding the matched component based on two realizations. At the next step, the methodology seeks for the highest absolute spatial correlation coefficients of the identified component in the remaining realizations factoring out all others. The procedure is repeated *M* times until *M* aligned components are found.

The next step involves the computation of the reproducibility index for each of the aligned components. This is done by constructing the histogram of the absolute spatial correlation coefficients of the upper triangle matrix of the CRCM. This histogram tends to be bimodal, as in general, we expect a low spatial correlation among most of the ICs and a high spatial correlation only for a few of them. A spatial correlation threshold is applied with the desired value lying in the valley of the histogram between the two modes (Yang et al. 2008). Finally, the reproducibility index is computed for each one of the aligned components. This is done by aggregating the supra-threshold absolute spatial correlation coefficients of the CRCM for each of the aligned components.

The last step of the algorithm is the ranking and averaging of the aligned components in descending order based on the reproducibility index. The selective averaging is applied so that the components are averaged if and only if, the given aligned component has at least one absolute spatial correlation coefficient above the threshold across realizations.

After applying RAICAR, the ICs are chosen via a cut-off threshold based on the reproducibility index (of each component) that indicates how consistent is the appearance of an IC across realizations.

Here, we have set $K=30$ realizations (same also in Yang et al. 2008); taking more realizations did not change the outcomes of the analysis. The spatial correlation threshold was chosen by localizing the minimum of the histogram of the absolute spatial correlation coefficients of the CRCM. This threshold was specified separately for each subject. The reproducible ICs were determined by calculating the reproducibility index. The cut-off threshold was set as the half of the maximum reproducibility index value possible $\frac{K(K-1)}{2}\cdot 0.5$ (this choice is the same with the one used in Yang et al. 2008). This cut-off threshold was set equal for all subjects.

Subjects with less than 20 reproducible ICs were excluded from further analysis as this number of components resulted in disconnected graphs. Thus, we ended up with 104 subjects out of which 57 were healthy controls and 47 schizophrenia patients.

### Construction of functional connectivity networks

For the construction of FCN, we used all combinations between five manifold learning algorithms, namely MDS, ISOMAP, diffusion maps, LLE , kPCA and two widely used metrics, namely the cross correlation (Anderson and Cohen 2013; Meszlényi et al. 2017; Hyde and Jesmanowicz 2012) and the Euclidean distance (Sipola et al. 2013; Venkataraman et al. 2009; Goutte et al. 1999).

#### Construction of FCN based on cross correlation

For every pair of the associated time courses of the ICs, say ${\mathbf {A}}_i$ and ${\mathbf {A}}_{j}$, the cross correlation function (CCF) over *l* time lags reads:

2 $$\begin{aligned} CCF({\mathbf {A}}_{i},{\mathbf {A}}_{j},l)=\frac{E[({\mathbf {A}}_{i,t+l}-\overline{{\mathbf {A}}}_{i})({\mathbf {A}}_{j,t}-\overline{{\mathbf {A}}}_{j})]}{\sqrt{E[({\mathbf {A}}_{i,t}-\overline{{\mathbf {A}}}_{i})^2]E[({\mathbf {A}}_{j,t}-\overline{{\mathbf {A}}}_{j}})^2]}, \end{aligned}$$

where *l* is the time lag, and $\overline{{\mathbf {A}}}_{i}$ is the mean value of the whole time series. Here, we considered a maximum of three time lags (as in Anderson and Cohen (2013)).

For the construction of the connectivity/ correlation matrices, we used a pseudo-distance measure $d_{c}$ defined as (see also Anderson and Cohen (2013)):

3 $$\begin{aligned} d_{c}({\mathbf {A}}_{i},{\mathbf {A}}_{j})=1-\max _{l=0,1,2,3}(|CCF({\mathbf {A}}_{i},{\mathbf {A}}_{j},l)|). \end{aligned}$$

The resulting (dis)similarity matrices are fully connected and therefore are hardly comparable between subjects (see the discussion in Anderson and Cohen 2013). Thus, here as a standard practice, (and in all other algorithms described below), we applied thresholding to the (dis)similarity matrices in order to keep the strongest connections of the derived functional connectivity matrices. In order to factor out the influence of the variable network density on the computation and comparison of graph-theoretic measures across groups (van den Heuvel et al. 2017), we have implemented the approach of proportional thresholding (PT) (van den Heuvel et al. 2017). In particular, we considered a range of levels of PT from 20 to 70% with a step of 2%. Below the threshold of 20%, some graphs became too fragmented (i.e the graph breaks down to subgraphs with a small number of nodes), while thresholds above the 70% comprised of edges with low functional connections (see in Algunaid et al. 2018). Despite the fact that there is no consensus upon the ideal range of PT in the literature [studies typically report a PT range of 10–50% of the strongest edges (Algunaid et al. 2018; Xiang et al. 2020)], we decided to include a wide range of thresholds to assess the performance of each method/combination used. Using a narrow range of thresholds could result to incomplete or misleading results (Garrison et al. 2015).

Finally, if a graph was fragmented after thresholding, the largest component (i.e. the subgraph with the largest number of nodes) was used for further analysis.

#### Construction of FCN based on the Euclidean distance

The Euclidean distance is used in many studies to assess (dis)similarities between fMRI time series (Sipola et al. 2013; Venkataraman et al. 2009; Goutte et al. 1999). For time series associated with the independent spatial maps, ${\mathbf {A}}_{i}$ and ${\mathbf {A}}_{j}$, the Euclidean distance reads:

4 $$\begin{aligned} L_{2}({\mathbf {A}}_{i},{\mathbf {A}}_{j})=\sqrt{\sum _{t=1}^{T}(A_{i,t}-A_{j,t})^2}. \end{aligned}$$

For the construction of FCN, PT was applied to the Euclidean distance matrices for each individual over the range of 20–70%.

### Construction of FCN with manifold learning algorithms

Below, we present how MDS, ISOMAP, diffusion maps, kernel PCA and LLE can be exploited to construct (embedded) FCN.

#### Construction of FCN with MDS

The classical multidimensional scaling (Kruskal 1964) is a form of dimensionality reduction that can be used to find similarities between pairs of objects in a low-dimensional (embedded) space. Given a set of *M* objects/observables ${\mathbf {x}}_1,{\mathbf {x}}_{2},\dots ,{\mathbf {x}}_{M} \in {\mathbf {R}}^N$, MDS produces a low-dimensional data representation ${\mathbf {y}}_{1},{\mathbf {y}}_{2},\dots ,{\mathbf {y}}_{M} \in {\mathbf {R}}^p, p \ll N$ minimizing the objective function:

5 $$\begin{aligned} \sum \limits _{i,j,\ i \ne j } \Big ( \Vert {\mathbf {x}}_{i}-{\mathbf {x}}_{j}\Vert -d({\mathbf {x}}_i,{\mathbf {x}}_j)\Big )^2, \end{aligned}$$

where $d({\mathbf {x}}_i,{\mathbf {x}}_j)$ is the (dis)similarity obtained (eg. by any (dis)similarity measure of choice, however, when using the euclidean distance, the classical MDS produces a linear mapping equivalent to PCA) between all pairs of points ${\mathbf {x}}_1,{\mathbf {x}}_{2},\dots ,{\mathbf {x}}_{M} \in {\mathbf {R}}^N$. In our case, the observables ${\mathbf {x}}_i$ are the amplitudes of the spatial ICs ${\mathbf {A}}_{i, \ i=1,\ldots M} \in {\mathbf {R}}^N$. Here, $N=150$ (number of time points).

The coordinates of the embedded manifold ${\mathbf {y}}_{1},{\mathbf {y}}_{2},\dots ,{\mathbf {y}}_{M}$ are given by:

6 $$\begin{aligned}{}[{\mathbf {y}}_1,\dots ,{\mathbf {y}}_M]= \varvec{\Lambda }_{p\times p}\cdot {\mathbf {V}}^{T}_{p \times M}. \end{aligned}$$

$\varvec{\Lambda }_{p\times p}$ contains the square roots of the *p* largest eigenvalues, and ${\mathbf {V}}^{T}_{p \times M}$ are the corresponding eigenvectors of the matrix:

7 $$\begin{aligned} {\mathbf {B}}=-\frac{1}{2}{\mathbf {H}}{\mathbf {D}}^2 {\mathbf {H}}. \end{aligned}$$

${\mathbf {H}}_{M \times M}$ is the centering matrix defined as:

8 $$\begin{aligned} {\mathbf {H}}={\mathbf {I}}- \frac{1}{M} {\mathbf {1}} \cdot {\mathbf {1}}^T, \quad \mathbf {1}=\begin{bmatrix}1\\1\\\vdots\\1 \end{bmatrix}_{M\times 1}. \end{aligned}$$

The dimensionality reduction of the original data ${\mathbf {X}}={\mathbf {x}}_{1},{\mathbf {x}}_{2},\dots ,{\mathbf {x}}_{M} \in {\mathbf {R}}^N$ yields the embedding of ${\mathbf {Y}}={{\mathbf {y}}_{1},{\mathbf {y}}_{2},\dots ,{\mathbf {y}}_{M} \in {\mathbf {R}}^p}$, $p \ll N$. Here, for the construction of the embedded FCN, we produced distance matrices $\mathbf {D_{Y}}$ of size $M \times M$. For the implementation of the MDS algorithm, we used the “cmdscale” function contained in the package “Stats” in the R free Software Environment (Team 2014).

#### Construction of FCN using ISOMAP

ISOMAP is a non-linear manifold learning algorithm that given a set of *M* objects/observables ${\mathbf {x}}_{1},{\mathbf {x}}_{2},\dots ,{\mathbf {x}}_{M} \in {\mathbf {R}}^N$ produces a low-dimensional data representation ${\mathbf {y}}_{1},{\mathbf {y}}_{2},\dots ,{\mathbf {y}}_{M} \in {\mathbf {R}}^p$, $p \ll N$ minimizing the objective function:

9 $$\begin{aligned} \sum \limits _{i,j, \ i \ne j } \Big ( \ d_G({\mathbf {x}}_i,{\mathbf {x}}_j)- d({\mathbf {x}}_{i},{\mathbf {x}}_{j})\Big )^2, \end{aligned}$$

where $d_G({\mathbf {x}}_i,{\mathbf {x}}_j)$ is the shortest path (geodesic distance) and $d({\mathbf {x}}_i,{\mathbf {x}}_j)$ is the (dis)similarity obtained (by any (dis)similarity measure of choice) between all pairs of points ${\mathbf {x}}_{1},{\mathbf {x}}_{2},\dots ,{\mathbf {x}}_{M} \in {\mathbf {R}}^N$.

In our case, the observables ${\mathbf {x}}_i$ are the amplitudes of the spatial ICs ${\mathbf {A}}_{i, \ i=1,\ldots M} \in {\mathbf {R}}^N$.

The above minimization problem is solved as follows (Tenenbaum et al. 2000):

- Construct a graph ${\mathbf {G}}=(V,E)$, where the vertices *V* are the ICs ${\mathbf {A}}_i$; its links *E* are created by using either the *k*-nearest neighbors algorithm or a fixed distance between nodes, known as the $\epsilon$ distance. For example, a link between two ICs is created if $d_{i,j}\equiv d({\mathbf {A}}_i,{\mathbf {A}}_j)< \epsilon \ , \ \forall \ i \ne j$. Here, we used the *k* nearest neighbours algorithm with $k=3,4,5,6$ (Tenenbaum et al. 2000). A general rule of thumb is to select *k* as the square root of the number of samples (here the number of ICs per subject). In our study the number of samples varied over subjects in the range of 20–40. Additionally, Anderson and Cohen (2013) use a similar approach by selecting *k* as 10% of the number of nodes. For $k=2$, we had some graphs that were disconnected and so we chose not to include this value. Set the weight $w_{i,j}$ of the link (if any) between ${\mathbf {A}}_i,{\mathbf {A}}_j$ as $w_{i,j}= \frac{1}{d({\mathbf {A}}_i,{\mathbf {A}}_j)}$. If there is not a link set: $w_{i,j}=0$.
- Approximate the embedded manifold by estimating the shortest path (geodesic distance) $d_G ({\mathbf {A}}_i,{\mathbf {A}}_j)$ for each pair of nodes based on the distances $d_{i,j}$; this step can be implemented for example using the Dijkstra algorithm (Dijkstra 1959). This procedure results in a matrix, ${\mathbf {D_G}}$ whose elements are the shortest paths:
  10 $$\begin{aligned} D_{G_{ij}}\equiv & {} d_G ({\mathbf {A}}_i,{\mathbf {A}}_j)\nonumber \\&= min \big \{ d_{i,j},d_{i,k}+d_{k,j} \big \}, \,k=1,2,\dots ,M \quad k \ne i,j. \end{aligned}$$
- Estimate the coordinates of the low-dimensional (embedded) manifold ${\mathbf {y}}_1,{\mathbf {y}}_2,\dots ,{\mathbf {y}}_M$ exploiting the MDS algorithm (Kruskal 1964) on the geodesic distance matrix ${\mathbf {D_G}}$.

Here, for the implementation of the ISOMAP algorithm, we used the package “vegan” (Oksanen et al. 2007) in the R free software environment (Team 2014).

#### Construction of FCN using diffusion maps

Diffusion maps (Coifman and Lafon 2006) is a non-linear manifold learning algorithm that given a set of *M* objects/observables ${\mathbf {X}}={\mathbf {x}}_{1},{\mathbf {x}}_{2},\dots ,{\mathbf {x}}_{M} \in {\mathbf {R}}^N$ produces a low-dimensional representation ${\mathbf {Y}}={{\mathbf {y}}_{1},{\mathbf {y}}_{2},\dots ,{\mathbf {y}}_{M}} \in {\mathbf {R}}^p$, $p \ll N$, addressing the diffusion distance among data points as the preserved metric (Nadler et al. 2006). The embedding of the data in the low-dimensional space is obtained by the projections on the eigenvectors of a normalized Laplacian graph (Belkin and Niyogi 2003). The diffusion maps algorithm can be described in a nutshell in the following steps:

- Construction of the affinity matrix ${\mathbf {W}}_{M \times M}$, here *M* is the number of ICs for each subject. The elements $W_{ij}$ represent the weighted edges connecting nodes *i* and *j* using the so-called heat kernel:
  11 $$\begin{aligned} W_{i,j}= exp\left( - \frac{d({\mathbf {x}}_{i},{\mathbf {x}}_{j})^2}{\sigma } \right) , \end{aligned}$$
  where ${\mathbf {x}}_i$ is a *N*-dimensional point (here, N=150), $d({\mathbf {x}}_i,{\mathbf {x}}_j)$ are the (dis)similarities obtained (by any dissimilarity measure of choice) between all pairs of points ${\mathbf {x}}_{1},{\mathbf {x}}_{2},\dots ,{\mathbf {x}}_{M} \in {\mathbf {R}}^N$ and $\sigma$ is an appropriately chosen parameter which can be physically described as a scale parameter of the heat kernel (Coifman and Lafon 2006). The heat kernel ${\mathbf {W}}$ satisfies two important properties, the one of symmetry and the other of the positive semi-definite matrix. The latter property is crucial and allows the interpretation of weights as scaled probabilities of “jumping” from one node to another. The parameter $\sigma$ of the neighborhood size is data-dependent and here, it was determined by finding the linear region in the sum of all weights in ${\mathbf {W}}$, say $S_w$, using different values of $\sigma$ (Singer et al. 2009; Sipola et al. 2013). $S_w$ is calculated through the formula:
  12 $$\begin{aligned} S_w=\sum \limits _{i}^M\sum \limits _{j}^M{W_{ij}}, \end{aligned}$$
  In order to use a single value of $\sigma$ for all participants, we computed a super-distribution of the sum of weights across subjects (taking the median value of the distributions) using different values of $\sigma$. Thus, we considered values of $\sigma$ lying in the linear region of the super-distribution. Because the sum of weights is a sigmoidal function of $\sigma$, we found the value of $\sigma$ where the maximum slope is attained. We then considered as “linear region”, the neighborhood of $\sigma$ with small bidirectional changes around that point (accounting to 5 % of the maximum slope).
- Formulation of the diagonal $M \times M$ normalization matrix ${\mathbf {K}}$ along with the diffusion matrix ${\mathbf {P}}$:
  13 $$\begin{aligned} K_{ii}&= \sum _{j=1}^{M} W_{ij}, \end{aligned}$$
  14 $$\begin{aligned} {\mathbf {P}}&= {\mathbf {K}}^{-1}{\mathbf {W}}. \end{aligned}$$
  Each element of the symmetric and normalized diffusion matrix ${\mathbf {P}}$ reflects the connectivity between two data points ${\mathbf {x}}_{i}$ and ${\mathbf {x}}_{j}$. As an analogy, this connectivity can be seen as the probability of “jumping” from one point to another in a random walk process. Consequently, raising ${\mathbf {P}}$ to a power of *t* can be thought of as a diffusion process. As the number of *t* increases, paths with low probability tend to zero, while the connectivity between paths with high probability remains high enough governing the diffusion process (Coifman and Lafon 2006). Thus, the algorithm of diffusion maps preserves the diffusion distance among points in a low-dimensional Euclidean space. The diffusion distance is closely related to the diffusion matrix ${\mathbf {P}}$; for two distinct points ${\mathbf {x}}_{i}$, ${\mathbf {x}}_{j}$ and for specific time instance *t* is defined as (De la Porte et al. 2008):
  15 $$\begin{aligned} D_{t}({\mathbf {x}}_{i},{\mathbf {x}}_{j})=\sum _{m}|P_{im}^{t}-P_{mj}^{t}|^2. \end{aligned}$$
  Unlike the geodesic distance, the diffusion distance is robust to noise perturbations, as it sums over all possible paths (of *t* steps) between points (Coifman and Lafon 2006).
- Construction of the conjugate matrix
  16 $$\begin{aligned} {\overline{\mathbf {P}}}= {\mathbf {K}}^{1/2}{\mathbf {P}}{\mathbf {K}}^{-1/2}, \end{aligned}$$
  substituting Eq.(14) to Eq.(16) we get
  17 $$\begin{aligned} {\overline{\mathbf {P}}}= {\mathbf {K}}^{-1/2}{\mathbf {W}}{\mathbf {K}}^{-1/2}. \end{aligned}$$
  This is the so-called graph Laplacian matrix (Belkin and Niyogi 2003). The matrix ${\mathbf {\ P}}$ is adjoint to the symmetric matrix ${\overline{\mathbf {P}}}$. Thus, ${\mathbf {\ P}}$ and ${\overline{\mathbf {P}}}$ share the same eigenvalues (Nadler et al. 2008).
- Singular Value Decomposition (SVD) of ${\overline{\mathbf {P}}}$ yields
  18 $$\begin{aligned} {\overline{\mathbf {P}}}={\mathbf {U}}\varvec{\Lambda } {\mathbf {U}}^{*}, \end{aligned}$$
  where $\varvec{\Lambda }$ is a diagonal matrix containing the *M* eigenvalues of ${\mathbf {P}}$ and ${\mathbf {U}}$ the eigenvectors of ${\overline{\mathbf {P}}}$. The eigenvectors ${\mathbf {V}}$ of ${\mathbf {P}}$ can be found by (Nadler et al. 2008):
  19 $$\begin{aligned} {\mathbf {\ V}}={\mathbf {K}}^{-1/2}{\mathbf {U}}. \end{aligned}$$
- By taking out the trivial eigenvalue $\lambda =1$ of the matrix $\varvec{\Lambda }$ and the corresponding eigenvector contained in ${\mathbf {V}}$, the coordinates of the low dimensional embedded manifold ${\mathbf {y}}_{1},{\mathbf {y}}_{2},\dots ,{\mathbf {y}}_{M}$ are given by:
  20 $$\begin{aligned}{}[{\mathbf {y}}_1,\dots ,{\mathbf {y}}_M]= \varvec{\Lambda }_{p\times p}\cdot {\mathbf {V}}^{T}_{p \times M}, \end{aligned}$$
  where $\varvec{\Lambda }_{p\times p}$ contains the *p* largest eigenvalues, and ${\mathbf {V}}^{T}_{p \times M}$ are the corresponding eigenvectors of the diffusion matrix ${\mathbf {P}}$.

For the implementation of the above algorithm, we used the package “diffusionMap” (Richards 2014) in the R free software environment (Team 2014).

#### Construction of FCN using kernel principal component analysis

Kernel PCA (Schölkopf et al. 1997) is an extension of the linear PCA (Jollife 2002) to produce a non-linear mapping (Muller et al. 2001) of the data. Given a set of *M* objects/observables ${\mathbf {X}}={\mathbf {x}}_{1},{\mathbf {x}}_{2},\dots ,{\mathbf {x}}_{M} \in {\mathbf {R}}^N$, kPCA produces a low-dimensional representation ${\mathbf {Y}}={{\mathbf {y}}_{1},{\mathbf {y}}_{2},\dots ,{\mathbf {y}}_{M}} \in {\mathbf {R}}^p$, $p \ll N$. The standard procedure follows three simple steps:

- Introduce a non-linear mapping ${\mathbf {X}} \rightarrow \phi ({\mathbf {x}})$.
- Calculate the covariance matrix ${\mathbf {C}}=E \Big \{ \phi ({\mathbf {x}})\phi ({\mathbf {x}})^T \Big \}$.
- Solve the eigenvalue problem ${\mathbf {C}}{\mathbf {u}}=\lambda {\mathbf {u}}$.
- project ${\mathbf {C}}$ on the eigenvectors that correspond to the largest eigenvalues (that account for most of the variance).

Using the so called “kernel” trick, we can rule out the actual mapping and dot product operations (Schölkopf et al. 1997). Instead, we simply have to estimate a kernel function. Here, we use the Gaussian kernel (following notation in Tsatsishvili et al. 2018):

21 $$\begin{aligned} K_{i,j}= exp\left( - \frac{d({\mathbf {x}}_{i},{\mathbf {x}}_{j})^2}{2\gamma ^2}\right) . \end{aligned}$$

where $d({\mathbf {x}}_i,{\mathbf {x}}_j)$ are the (dis)similarities obtained (by any dissimilarity measure of choice) between all pairs of points ${\mathbf {x}}_{1},{\mathbf {x}}_{2},\dots ,{\mathbf {x}}_{M} \in {\mathbf {R}}^N$ and $\gamma$ is a free parameter of the Gaussian kernel. For each subject, we considered $\gamma$ to be the median of the minimum values of distances among data points (as proposed also in Tsatsishvili et al. (2018)). As ${\mathbf {K}}$ is not guaranteed to be centered, it is required to “centralize” *K* using the centering matrix ${\mathbf {H}}$ :

22 $$\begin{aligned} \mathbf {K}^{\prime }={\mathbf {H}}{\mathbf {K}} {\mathbf {H}}. \end{aligned}$$

Next we need to solve the eigenvalue problem:

23 $$\begin{aligned} \mathbf {K}^{\prime } \cdot {\mathbf {V}}=\varvec{\Lambda } \cdot {\mathbf {V}} \end{aligned}$$

where ${\mathbf {V}}$ contains the eigenvectors and the diagonal matrix $\varvec{\Lambda }$ contains the eigenvalues of $\mathbf {K}^{\prime }$.

The coordinates of the embedded manifold ${\mathbf {y}}_{1},{\mathbf {y}}_{2},\dots ,{\mathbf {y}}_{M}$ are finally obtained by projecting the centered kernel matrix ${\mathbf {K}^{\prime }}$ onto its eigenvectors that correspond to the *p* largest eigenvalues:

24 $$\begin{aligned}{}[{\mathbf {y}}_1,\dots ,{\mathbf {y}}_M]= ({\mathbf {K}^{\prime }}_{M\times M}\cdot {\mathbf {V}}_{M \times p})^{T}. \end{aligned}$$

For the implementation of the kernel PCA, we used the package ”kernlab” (Karatzoglou et al. 2004) in the R free software environment (Team 2014).

#### Construction of FCN using locally linear embedding

Locally Linear Embedding (LLE) (Roweis and Saul 2000) is a non-linear manifold learning technique that given a set of *M* objects/observables ${\mathbf {X}}={\mathbf {x}}_{1},{\mathbf {x}}_{2},\dots ,{\mathbf {x}}_{M} \in {\mathbf {R}}^N$ produces a low-dimensional representation ${\mathbf {Y}}={{\mathbf {y}}_{1},{\mathbf {y}}_{2},\dots ,{\mathbf {y}}_{M}} \in {\mathbf {R}}^p$, $p \ll N$, that preserves the local topology (i.e the distance between neighbouring data points). The LLE assumes that even if the high dimensional data points lie on a highly non-linear manifold, the manifold can be still considered as locally linear. Provided that the manifold is well sampled, then we would expect that every data point has neighbours that lie on or close to a linear patch of the global manifold. According to this assumption, LLE approximates every data point in a low-dimensional space by calculating a weighted linear combination of its neighbours. Thus, LLE yields a low dimensional representation of data by learning the global structure, from local relationships (Roweis and Saul 2000).

The main procedure can be described in three steps:

- Find the nearest neighbours of data points by using either the *k*-nearest neighbors algorithm or a fixed distance between data points, known as the $\epsilon$ distance. Here, we used the *k*-nearest neighbors algorithm.
- Compute the weights ${\mathbf {W}}_{i}$ that best reconstruct linearly each data point ${\mathbf {x}}_{i}$ from its neighbours with respect to the following optimization problem:
  25 $$\begin{aligned} {\mathbf {W}}_{i}=\hbox {argmin} \left\| {\mathbf {x}}_{i}-\sum \limits _{k} w_{ik}{\mathbf {x}}_{k}\right\| ^2. \end{aligned}$$
  Constraints to the above minimization scheme include: $w_{ik}=0$, if ${\mathbf {x}}_{k}$ is not a neighbour of ${\mathbf {x}}_{i}$ (each data point is reconstructed only from its neighbours), $\sum \nolimits _{k}w_{ik}=1$ (all weights of neighbouring points *k* sum to 1).
- Embedding coordinates ${\mathbf {y}}_{i}$ that best preserve the local structure of neighbourhoods of ${\mathbf {x}}_{i}$ in the low dimensional space are given by:
  26 $$\begin{aligned} {\mathbf {y}}_{i}=\hbox {argmin} \left\| {\mathbf {y}}_{i}-\sum \limits _{k} w_{ik}{\mathbf {y}}_{k}\right\| ^2, \end{aligned}$$
  with respect to ${\mathbf {y}}_i \in {\mathbf {R}}^p$, $p \ll N$. For the problem to be well posed, the following constraints are set:
  27 $$\begin{aligned} \dfrac{1}{M} \sum \limits _{i} \mathbf {y}_{i}\mathbf {y}_{i}^{T}={\mathbf {I}} , \quad \sum \limits _{i} \mathbf{y}_{i}=\mathbf{0} \end{aligned}$$
  To find the embedding coordinates, we construct ${\mathbf {W}^{\prime }}={\mathbf {(I-W)}}^{T} {\mathbf {(I-W)}}$ and solve the eigenvalue problem. Here, ${\mathbf {I}}$ is the identity matrix and *M* is the number of the eigenvalues of ${\mathbf {W}^{\prime }}$. The first constraint $\dfrac{1}{M} \sum \limits _{i} \mathbf {y}_{i}\mathbf {y}_{i}^{T}={\mathbf {I}}$ forces the embedding vectors to have unit covariances to avoid degenerate solutions (Roweis and Saul 2000), while the second constraint requires the coordinates to be centered at the origin. The eigenvectors of ${\mathbf {W}^{\prime }}$ are all solutions of ${\mathbf {Y}}$, but those correspond to the *p* smallest eigenvalues are the ones that minimize (26). The smallest eigenvalue of ${\mathbf {W}^{\prime }}$ will always be zero and it is discarded. The next $M-1$ eigenvalues can be used as the new dimensions of the transformed data. Estimation of the final dimensionality of the transformation can be made by selecting eigenvectors that correspond to the number of the smallest eigenvalues that form a cluster (Kayo 2006).

For the parameter *k*, we considered values of $k=2,\dots 10$. The number of nodes (for each subject) in this study varied in the range of 20–40. Thus, the largest *k* accounts for the 25–50% of the nodes of a subject. For larger values of *k* the LLE algorithm uses too many neighbours and each data point is no longer “locally” retrieved from its nearest neighbours.

Here, we employed a variant of the above LLE algorithm that takes as inputs only the pairwise distances (${\mathbf {D_{X}}}$) among the data points on the initial space (this extension of the LLE is thoroughly described in Saul and Roweis 2003). This was necessary as we wanted to test different metrics. For the implementation of this variant of the LLE algorithm, we modified the code offered by the “lle” package (Diedrich et al. 2012) in the R free software environment (Team 2014).

#### Choice of the embedding dimension

The embedding dimension was determined via the eigenspectrum of the final decomposition for every dimensionality reduction/manifold learning algorithm, as identified by the gap between the first few larger eigenvalues (smaller eigenvalues for the LLE) and the rest of the eigenspectrum. These first few eigenmodes capture most of the distance differences between data points and are able to represent and uncover intrinsic properties of the data structure (Nadler et al. 2008; Strange and Zwiggelaar 2014; Saul et al. 2006). In order to determine the embedding dimension for the methods described above, we considered the following steps: we sorted the eigenvalues in decreasing order $\lambda _{1} \ge \lambda _{2} \ge \lambda _{3}$ $\cdots \ge \lambda _{M}$ ($\lambda _{1}$ is discarded for diffusion maps and $\lambda _{M}$ for LLE). Then, for each subject, we calculated the pairwise differences $\lambda _{1}-\lambda _{2}$, $\lambda _{2}-\lambda _{3},\ldots$ $,\lambda _{M-1}-\lambda _{M}$. A large numerical gap between two elements of this sequence of pairwise differences indicates the dimension beyond which the relative contributions are redundant (or small contributions are made for the reconstruction of the embedded FCN).

### Graph-theoretic measures

We analyzed the topological properties of the binary FCN graphs on the basis of three fundamental graph measures for neuroscience, namely, the average path length, the global clustering coefficient, and the median degree (Stam and Reijneveld 2007; Khajehpour et al. 2019; Anderson et al. 2010; Parhizi et al. 2018). In particular, given a graph ${\mathbf {G}}=(V,E)$ with $g_{ij}$ representing the link (0: unconnected or 1: connected) from node *i* to node *j* and $k_i= \sum ^{}_{j,{j\ne i}}g_{ij}$ the degree of node i, the graph measures are computed as follows:

- The average path length is defined by: $L= \frac{1}{N_V(N_V-1)}\sum _{i \ne j}D_{G_{ij}}$, i.e. is the average number of steps along the shortest paths $D_{G_{ij}}$ for all possible pairs of the network nodes. This is a measure of the efficiency of information or mass transport on a network between all possible pairs of nodes.
- The global clustering coefficient is defined by: $C_g= \frac{\sum _{} t_c}{\sum _{} t}$, where *t* is a triplet and $t_c$ is a closed triplet. A triplet of a graph consists of three nodes that are connected by either open (i.e open triplet) or closed (i.e closed triplet) ties. In general, this measure indicates how random or structured a graph is (in our case, in terms of functional segregation).
- The median degree $M_{k}$ is the median value of the degree distribution of ${\mathbf {G}}$. This measure reflects how well connected is the “median” network node in terms of the number of links that coincide with it.

An extensive review of the definitions and the meaning of the above key graph-theoretic measures with respect to brain functional networks can be found in Rubinov and Sporns (2010), Stam and Reijneveld (2007), Bullmore and Sporns (2009).

The computations for the graph analysis were performed utilizing the “igraph” (Csardi and Nepusz 2006) package in the R free software environment (Team 2014).

### Classification/ Machine Learning algorithms

Classification was assessed using machine learning algorithms, namely Linear Support Vector Machines (LSVM), Radial Support Vector Machines (RSVM), Artificial Neural Networks (ANN) and k-Nearest Neighbours (k-NN) classification (for a brief description of the above algorithms and their parameter grids see the “Appendix”). The features that were considered for classification were the three key graph measures (as stated in “Graph-theoretic measures” section) which are the most frequently used in neuroscience (Stam and Reijneveld 2007; Khajehpour et al. 2019; Anderson et al. 2010; Parhizi et al. 2018; Bullmore and Sporns 2009). Our intention was not to implement a feature selection algorithm but to assess the efficiency of the methods based only on these three fundamental measures. All three measures were given as input to the classifiers. The classification algorithms were trained, validated and tested using a tenfold cross validation scheme which was repeated 100 times. Thus, we separated the data in ten distinct sub-samples; nine of them were used as training sets and one of them was used for validation purposes. This process was repeated 10 times leaving out each time a different sub-sample which served as a validation set. The whole procedure was repeated 100 times. The overall classification rate was determined via the computation of the average classification rate over all the repetitions of the tenfold cross validation for each model.

The average confusion matrix (over all repetitions of the tenfold cross validation) was also computed for each classification model. The confusion matrix is a $2\times 2$ (in the case of binary classification) square matrix containing all true positives *TP*, false positives *FP*, true negatives *TN* and false negatives *FN*. Here, we considered as positives *P* the schizophrenia cases and as negatives *N* the healthy control cases. Sensitivity (also called the True Positive Rate) and specificity (also called the True Negative Rate) are basic statistical measures for the assessment of binary classifications. The sensitivity *TPR* is given by $TPR= \frac{TP}{TP+FN}$, while the specificity *TNR* is given by $TNR= \frac{TN}{TN+FP}$. Here, sensitivity characterizes the ability of the classifier to correctly identify a schizophrenic subject, while specificity is the ability of the classifier to correctly identify a healthy subject.

Here, we used the algorithms contained in the package “caret” (Kuhn et al. 2008) in the R free software environment (Team 2014).

## Results

### Signal extraction via RAICAR methodology

**Fig. 1:**
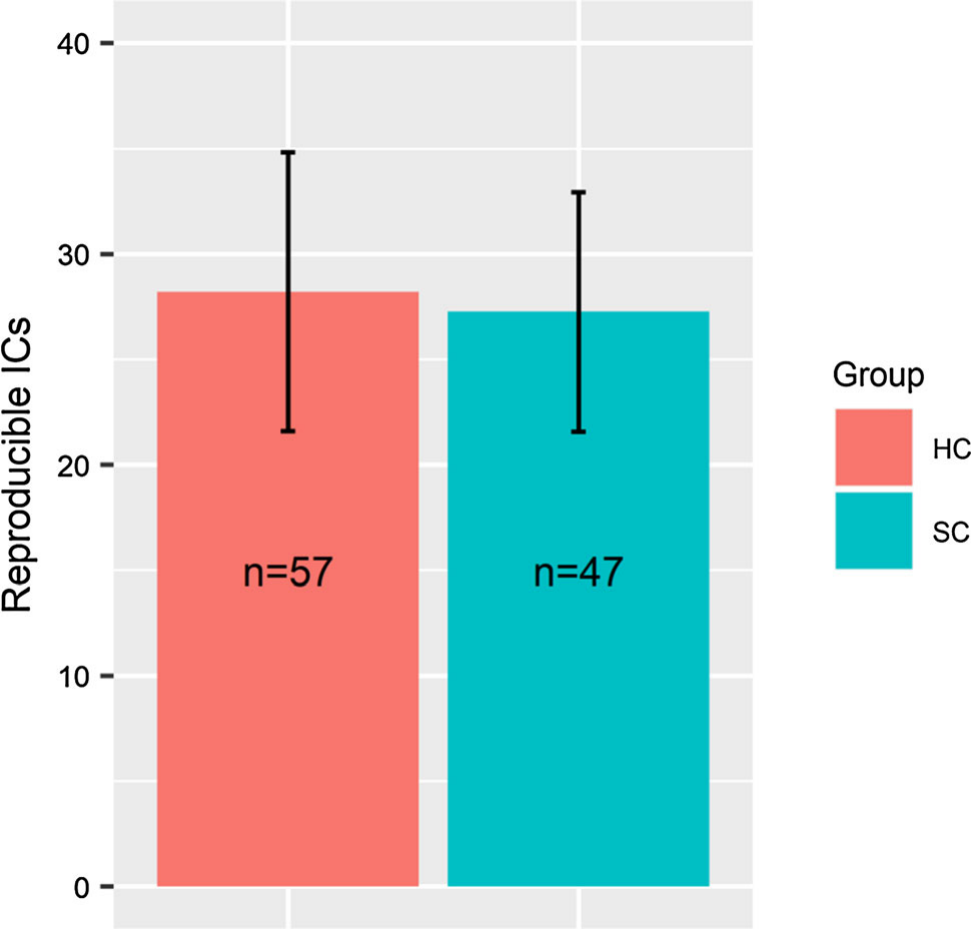
Reproducible components for the 57 healthy controls (red,“HC”) and 47 schizophrenic subjects (blue,“SC”) that resulted to 20 or more reproducible components. Each bar depicts the mean of the reproducible ICs extracted while the error bar represents the standard deviation for each group

Out of 72 patients only 47 of them exhibited 20 or more reproducible components. In comparison, 57 out of 74 healthy controls had 20 or more reproducible components. Figure 1 shows the mean of the reproducible components found for the group of healthy controls (red,“HC”) and schizophrenic subjects (blue,“SC”) along with the standard deviation (error bars). No statistically significant differences were found in the number of reproducible components between groups (Welch’s *t* test: *p* = 0.43).

### Classification performance using the cross correlation metric

In Table 1, we present the best classification accuracy, along with the corresponding sensitivity and specificity rate obtained for each manifold learning algorithm (see “Construction of FCN with manifold learning algorithms” section), PT point and classifier (see “Classification/ Machine Learning algorithms” section) with the cross correlation metric (see “Construction of FCN based on cross correlation” section). The optimal values of the parameters (i.e the embedding dimension *p* and method’s tuning parameter, see “Construction of FCN with manifold learning algorithms” section) for each method are also shown. At the end of Table 1, we also provide the results obtained by the “conventional” (thresholded) cross correlation matrix (see “Construction of FCN based on cross correlation” section). The best classification rate obtained for each method is marked with bold. Finally, the classification accuracy is reported along with the standard deviation (SD) over 100 repetitions of a tenfold cross validation scheme (see “Classification/ Machine Learning algorithms” section).

**Table 1:** Best classification rates over all manifold and machine learning methods using the cross correlation pseudo-distance measure $d_c$; optimal parameters are also shown for each method along with the corresponding PT, classifier, accuracy (Acc), sensitivity (Sens) and specificity (Spec) rate. Classifiers are noted as RSVM (Radial SVM), LSVM (Linear SVM), k-NN (k-NN classifier) and ANN (Artificial Neural Networks)

Method	Parameters	PT	Classifier	Acc ± SD (%)	Sens (%)	Spec (%)
*MDS*	*p* = 3	0.3	RSVM	**68.4** ± **1.3**	51.1	77.8
		0.36	LSVM	58.6 ± 1.7	28.8	78.9
		0.3	k-NN	63.2 ± 2.1	51	68.8
		0.3	ANN	63.6 ± 2.6	50.7	69.8
*ISOMAP*	*p* = 2, *k* = 5	0.24	RSVM	**74.4** ± **1.9**	69.4	73.1
		0.28	LSVM	64.4 ± 1.7	55.8	67.1
		0.24	k-NN	71 ± 2.1	62.9	72.7
		0.24	ANN	68.8 ± 3	63.1	68.6
*Diffusion maps*	*p* = 4, $\sigma$ = 0.325	0.52	RSVM	**79.3** ± **1.2**	74.1	77.9
		0.56	LSVM	72.2 ± 1.7	66.6	71.8
		0.52	k-NN	74.7 ± 1.4	72.3	71.5
		0.52	ANN	78.6 ± 2	74.1	76.8
*kPCA*	*p* = 4, $\gamma$ = 0.575	0.42	RSVM	**69.5** ± **1.6**	45.4	84.3
		0.3	LSVM	59.5 ± 2.4	19.1	88.6
		0.42	k-NN	67.1 ± 1.9	51.7	75.1
		0.42	ANN	69.4 ± 2.4	60.3	72
*LLE*	*p* = 4, *k* = 7	0.46	RSVM	68.3 ± 1.9	49	79.4
		0.46	LSVM	69.2 ± 1.4	48.6	81.3
		0.26	k-NN	66.1 ± 2.2	52.8	71.5
		0.46	ANN	**70.7** ± **1.3**	56	77.9
*Cross corr. matrix*	–	0.52	RSVM	69.5 ± 1.5	77.1	58.3
		0.52	LSVM	**71** ± **1.5**	75.8	61.9
		0.34	k-NN	67.2 ± 2.2	57.8	70.1
		0.52	ANN	68.8 ± 1.6	68.3	64.3

**Fig. 2:**
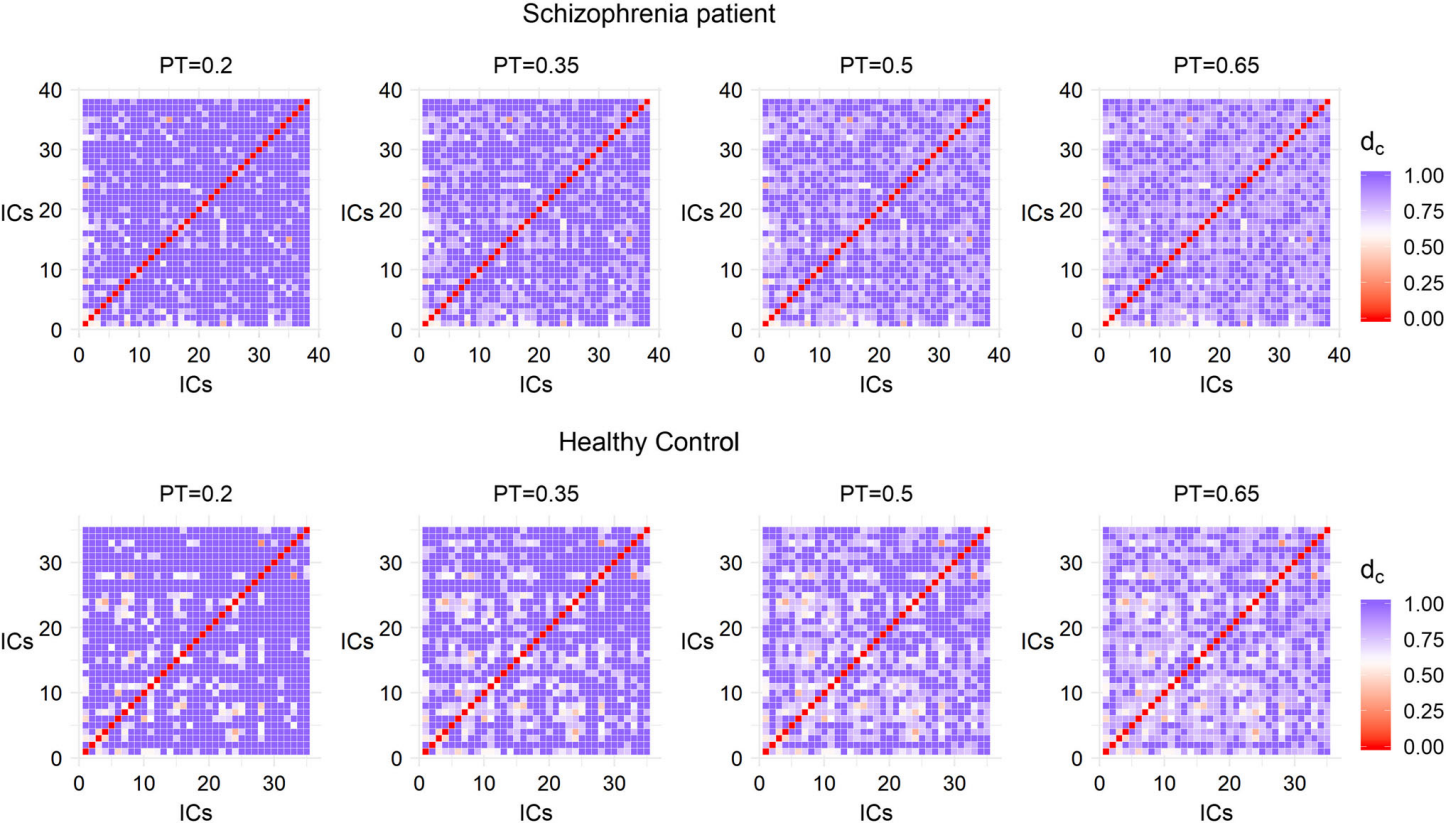
Visualization of the cross correlation matrix based on the pseudo-distance measure $d_{c}$ (see “Construction of FCN based on cross correlation” section) of a patient and a healthy control across different values of PT

Figure 2 provides a visualization of the cross correlation matrix of a patient and a healthy control across different values of PT (at 20%, 35%, 50% and 65% of the strongest edges). The metric used for the construction of the connectivity matrices is the pseudo-distance measure $d_{c}$ (see “Construction of FCN based on cross correlation” section) based on cross correlation. The lower the value of $d_{c}$ between 2 ICs, the more functionally connected they are.

**Fig. 3:**
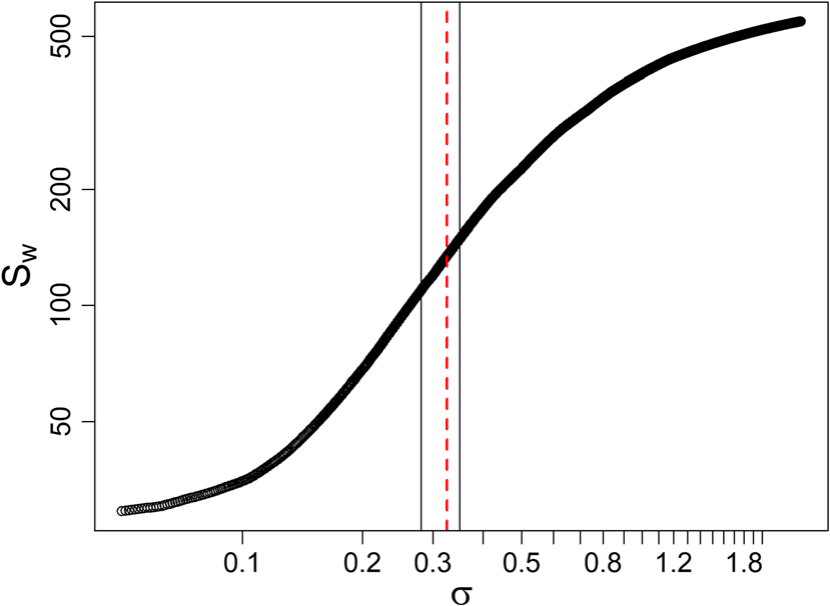
Super-distribution of all subjects of the sum of the weights (see Eq. 12). The red dashed vertical line shows the optimal $\sigma$ that was found to be $\sigma$ = 0.325. The other two vertical black lines bound the linear zone in which we investigated values of $\sigma$

Figure  3 shows the super-distribution of the sum of weights of all subjects with respect to different values of $\sigma$ used for the construction of the FCN with diffusion maps; the red dotted vertical line shows the optimal $\sigma$ (here, $\sigma$ = 0.325) while the black vertical lines bound the linear region ($\sigma \in (0.28,0.35)$). The results were robust to different choices of the time step of the diffusion maps algorithm, namely $t=0,1,2$.

**Fig. 4:**
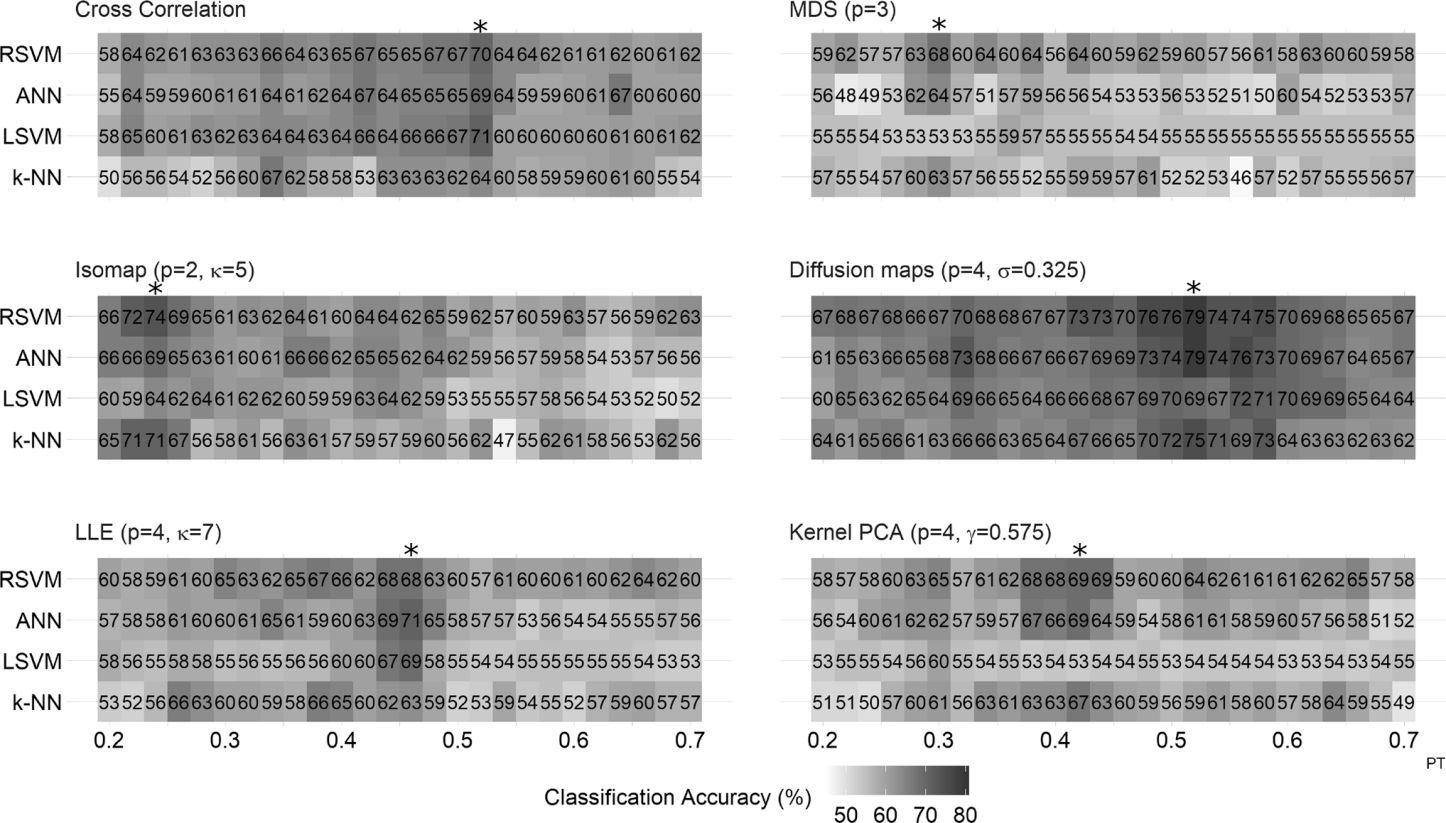
Overall classification performance for all thresholds (from 20 to 70% of the strongest edges with 2% as step) and classifiers using the optimal parameters. The metric used is the cross correlation based pseudo-distance measure $d_c$ (see “Construction of FCN based on cross correlation” section). The PT point with the best classification rate is marked with an asterisk “*”

Figure 4 depicts the classification rates for all manifold learning algorithms and the cross correlation matrix for all classifiers and PTs. The overall classification pattern over all PT points based on the optimal embedding dimension *p* and parameter for each method (marked on the top of each panel) is also shown. PT points with the best classification rates for each method are marked with an asterisk.

**Fig. 5:**
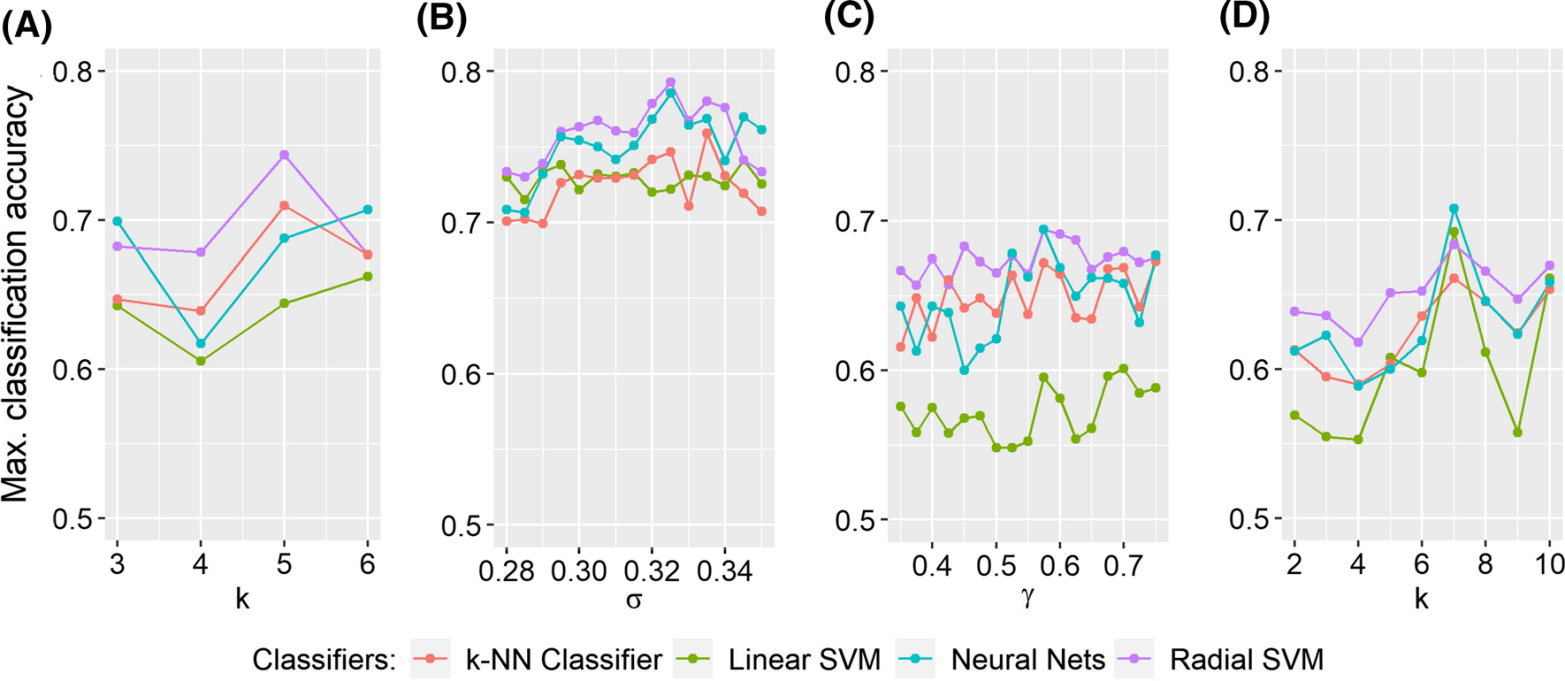
Classification performance of the parametric manifold learning techniques with respect to different parameter values. **A** ISOMAP (*k*), **B** diffusion maps ($\sigma$), **C** kPCA ($\gamma$), **D** LLE (*k*). The metric used is the pseudo-distance measure $d_c$ (see “Construction of FCN based on cross correlation” section) based on cross correlation

Figure 5 shows the classification performance of all parametric manifold learning techniques with respect to different values of the corresponding parameters. This figure shows how sensitive each method is (ISOMAP, diffusion maps, kPCA and LLE) to the changes of the parameter values. Details on the parameter grid selection are given in “Construction of FCN with manifold learning algorithms” section.

As shown, diffusion maps resulted in the best classification accuracy (79.3%, using RSVM and 52% PT), thus appearing more robust over a wide range of PTs (Fig. 4). With respect to the maximum classification accuracy obtained by diffusion maps, results were robust over a wide range of values of $\sigma \in (0.28,0.35)$ as it can be seen in Fig. 5B. All classifiers had maximum classification rates above 70%.

ISOMAP performed relatively better for lower PTs (Fig. 4), with the best classification rate being at 74.4% (using RSVM and 24% PT). Its performance was however sensitive to the choice of the number *k* of nearest neighbors; with $k=5$, we got a 74.4% classification accuracy while for $k=4$, we got a classification rate below 70% for all classifiers (Fig. 5A). In terms of the maximum classification rate, kPCA and LLE performed similarly to the cross correlation matrix (see Table 1). LLE was sensitive to the choice of *k* nearest neighbours; with $k=7$, LLE peaked at 70.7 % classification accuracy (using ANN and 52% PT ) while for most of the other values of *k*, the accuracy was below or nearly 65% (Fig. 5D). For kPCA and MDS the linear classifier (LSVM) consistently performed poorly resulting in most cases to a classification accuracy below 60% (Figs. 4, 5C). MDS was outperformed by all other methods (Table 1); only the RSVM’s performance was relatively robust against thresholding (Fig. 4). At most of the PT points, the performance of all the other classifiers was poor (the accuracy rates were below 60 %). On the other hand, diffusion maps and kPCA appeared more robust to the choice of parameter values (Fig. 5B, C).

For most of the manifold learning methods, the classifier that worked better was the RSVM classifier both in terms of the maximum classification rate but also with respect to different PT points (Table 1, Fig. 4). RSVM gave the highest maximum classification rate for the four out of the five manifold learning methods. Only for LLE, the ANN produced the highest accuracy rate at 70.7%.

**Fig. 6:**
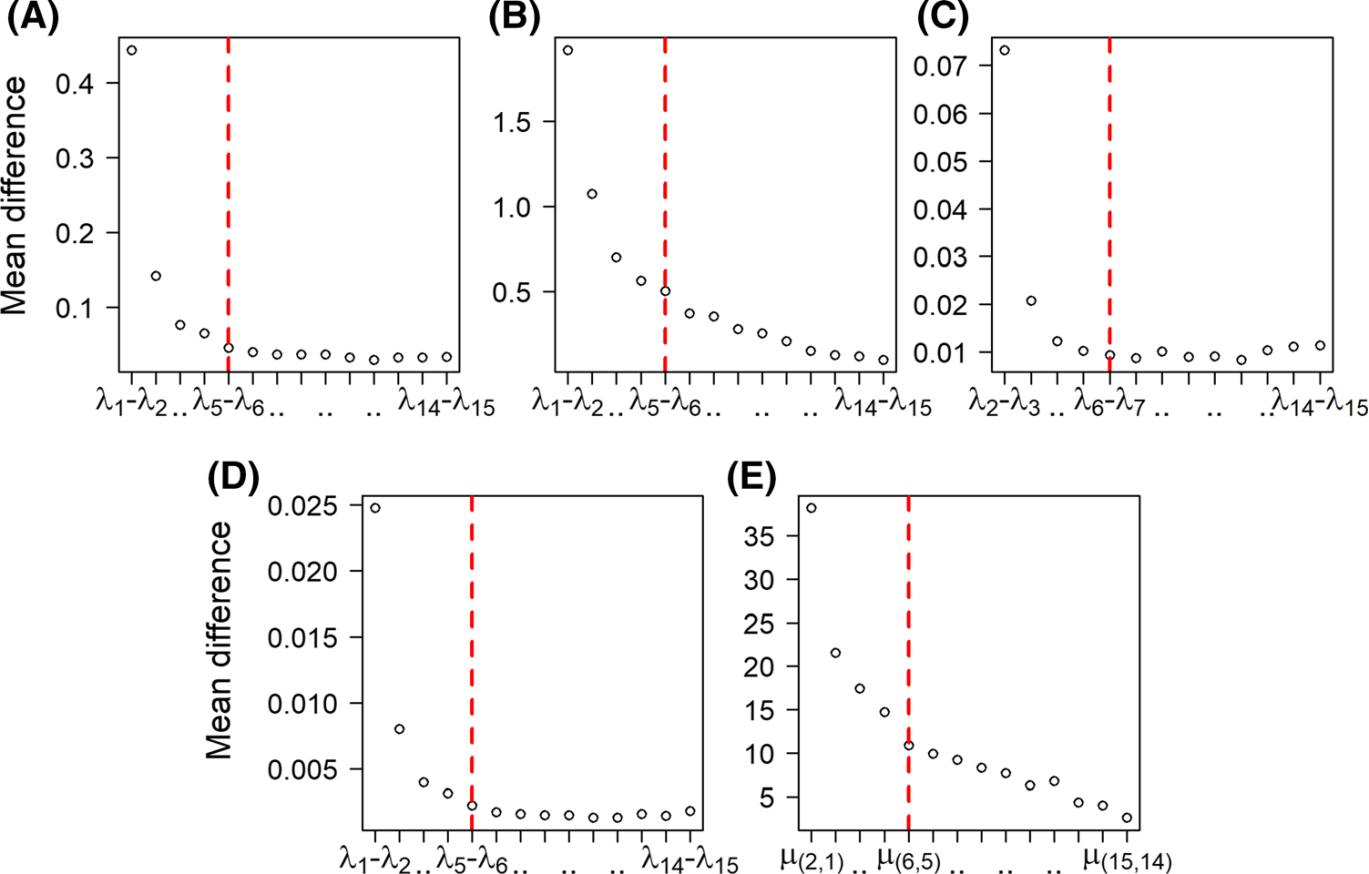
Mean differences of the 15 largest (smallest for the LLE) eigenvalues (see “Choice of the embedding dimension” section) for all manifold learning algorithms using the cross correlation-based pseudo distance measure $d_{c}$ (see “Construction of FCN based on cross correlation” section). **A** MDS (see in “Construction of FCN with MDS” section), **B** ISOMAP (see in “Construction of FCN using ISOMAP” section), **C** diffusion maps (see in “Construction of FCN using diffusion maps” section), **D** kPCA (see in “Construction of FCN using kernel principal component analysis” section), **E** LLE (see in “Construction of FCN using locally linear embedding” section) based on the optimal parameters. The red dashed vertical line marks the maximum number of dimensions considered in this study [i.e. the 5 dimensions (see “Choice of the embedding dimension” section)]. For the case of LLE, the function $\mu ({\mathbf {i,j}})=\frac{\mathbf {1}}{\lambda _{\mathbf {M-i}}-\lambda _{\mathbf {M-j}}}$ was used for visualization purposes as we are interested in the smallest eigenvalues (trivial eigenvalue $\lambda _{M}=0$ is discarded). (Color figure online)

Figure 6 shows the characteristic eigenspectrum of MDS, ISOMAP, diffusion maps, kPCA and LLE. As it is shown, in most of the cases there are three gaps: the first gap appears between the first eigenvalue and the rest of the spectrum, the second gap between the first two eigenvalues and the rest of the spectrum, and a third gap appears between the first four-five eigenvalues and the rest of the spectrum. Especially for the case of the LLE, we are interested in the smallest eigenvalues (see “Construction of FCN using locally linear embedding” section) of the final decomposition. For visualization purposes, we show the eigenspectrum in a similar manner using the function $\mu ({\mathbf {i,j}})=\frac{\mathbf {1}}{\lambda _{\mathbf {M-i}}-\lambda _{\mathbf {M-j}}}$, which is the inverse of the pairwise differences between the 15 smaller eigenvalues. In all cases, as dictated by the corresponding gaps, we considered a maximum of five eigendimensions for the construction of the embedded FCN (see “Choice of the embedding dimension” section).

### Classification performance using the Euclidean distance

The same analysis was performed for the Euclidean distance. The best classification rates using the Euclidean distance for all manifold learning methods and classifiers are presented in Table 2. At the end of Table 2, we present the results for the (thresholded) Euclidean matrix.

**Table 2:** Best classification rates over all manifold learning methods and classifiers with the use of the Euclidean distance $L_{2}$ (see “Construction of FCN based on the Euclidean distance” section); parameters, PT, classifier, accuracy (Acc), sensitivity (Sens) and specificity (Spec) rates. Classifiers are noted as RSVM (Radial SVM), LSVM (Linear SVM), k-NN (k-NN Classifier) and ANN (Artificial Neural Networks)

Method	Parameters	PT	Classifier	Acc ± SD (%)	Sens (%)	Spec (%)
*MDS*	*p* = 3	0.54	RSVM	**72.3** ± **1.7**	52.8	83.4
		0.36	LSVM	57.9 ± 2.5	26.6	79.6
		0.26	k-NN	65.7 ± 2.2	59.6	66.1
		0.44	ANN	64.9 ± 2.6	53.7	69.5
*ISOMAP*	*p* = 2, *k* = 5	0.68	RSVM	**72.9** ± **2**	55.8	81.9
		0.42	LSVM	54.6 ± 0.1	0	95.9
		0.68	k-NN	65 ± 2.3	47.7	74.7
		0.66	ANN	68.2 ± 1.8	47.4	80.4
*Diffusion maps*	*p* = 5, $\sigma$ = 110	0.66	RSVM	**68.8** ± **2.2**	57.7	73.1
		0.52	LSVM	62.9 ± 1.9	56.7	63.5
		0.26	k-NN	65.1 ± 2.5	63.3	61.7
		0.5	ANN	63.9 ± 2.6	71.4	53.2
*kPCA*	*p* = 5, $\gamma$ = 11.5	0.62	RSVM	67.1 ± 1.3	37.1	87.1
		0.28	LSVM	62.1 ± 1.6	56.8	62.9
		0.64	k-NN	62.7 ± 2.8	56.7	63.3
		0.62	ANN	**67.2** ± **1.6**	62.1	66.8
*LLE*	*p* = 3, *k* = 3	0.48	RSVM	**70.3** ± **2.6**	67.9	67.3
		0.26	LSVM	70.2 ± 1.4	68.9	66.4
		0.28	k-NN	65.3 ± 2.7	59.4	65.4
		0.26	ANN	70 ± 2	64.3	70
*Euclidean matrix*	–	0.64	RSVM	71.6 ± 1.7	60.1	76.1
		0.58	LSVM	59.2 ± 2.5	62.5	52.3
		0.64	k-NN	**72** ± **2.2**	67.5	70.6
		0.70	ANN	62.1 ± 2.6	60.4	59.1

**Fig. 7:**
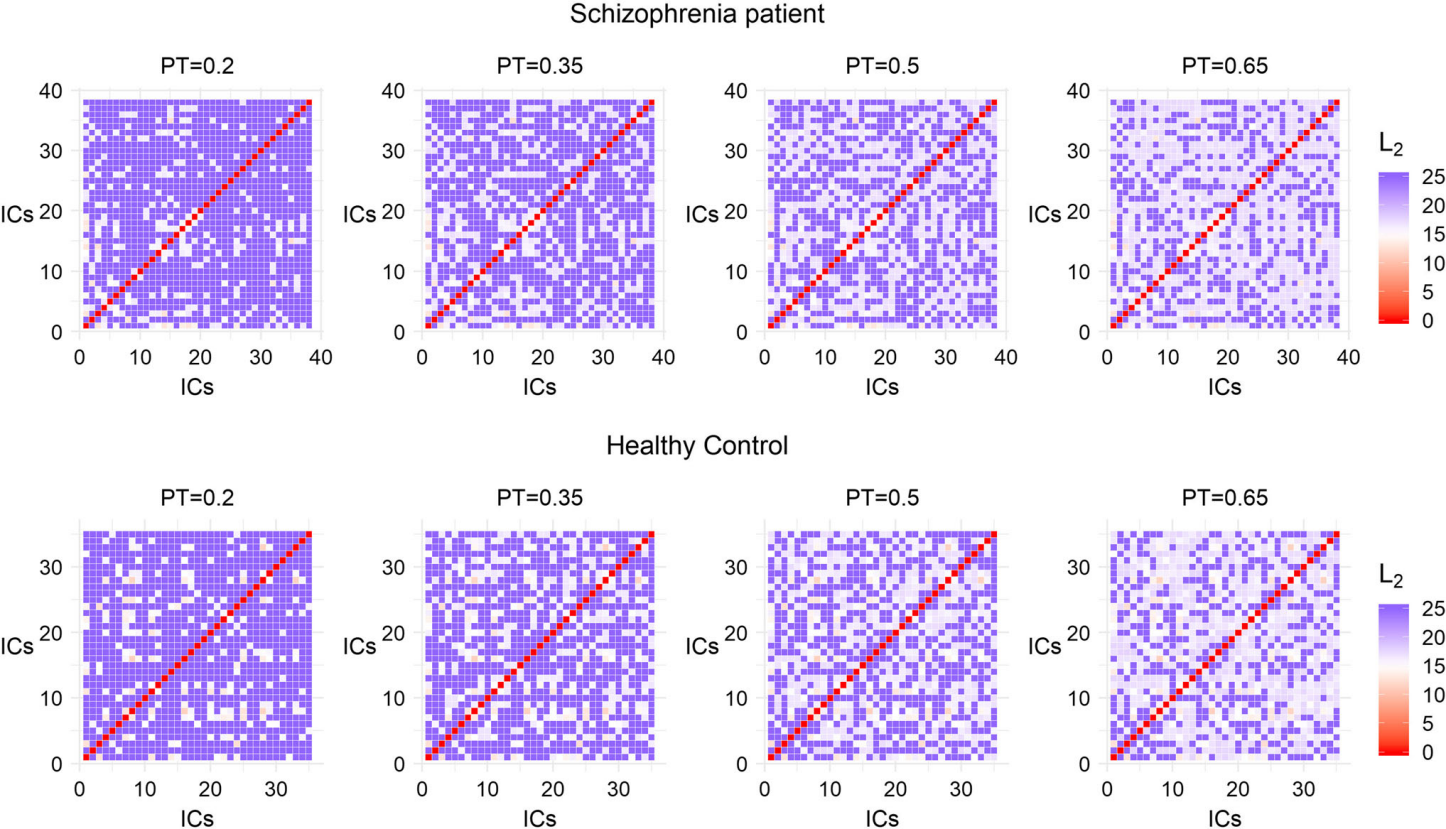
Visualization of the connectivity matrix constructed with the Euclidean distance $L_{2}$ (see “Construction of FCN based on the Euclidean distance” section) of a patient and a healthy control for different values of PT

Figure 7 provides a visualization of the connectivity matrices for the same patient and the same healthy control of Fig. 2 across different values of PT (at 20%, 35%, 50% and 65% of the strongest edges). The metric used is the Euclidean distance $L_{2}$ (see “Construction of FCN based on the Euclidean distance” section).

**Fig. 8:**
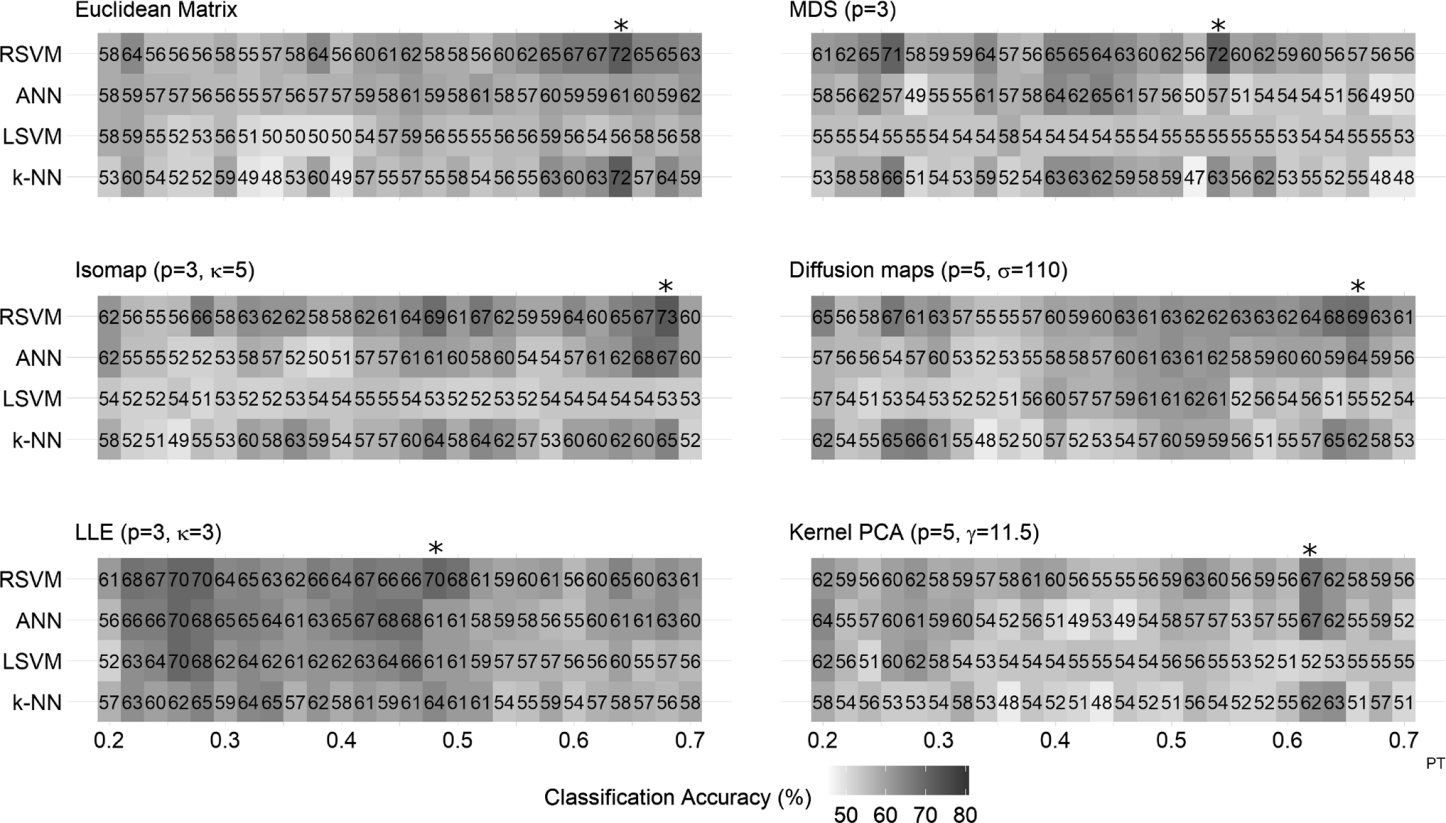
Classification performance using the Euclidean distance $L_{2}$ (see “Construction of FCN based on the Euclidean distance” section) for all thresholds (from 20 to 70% of the strongest edges with 2% as step) and classifiers. The PT point with the best classification rate is marked with an asterisk “*”

**Fig. 9:**
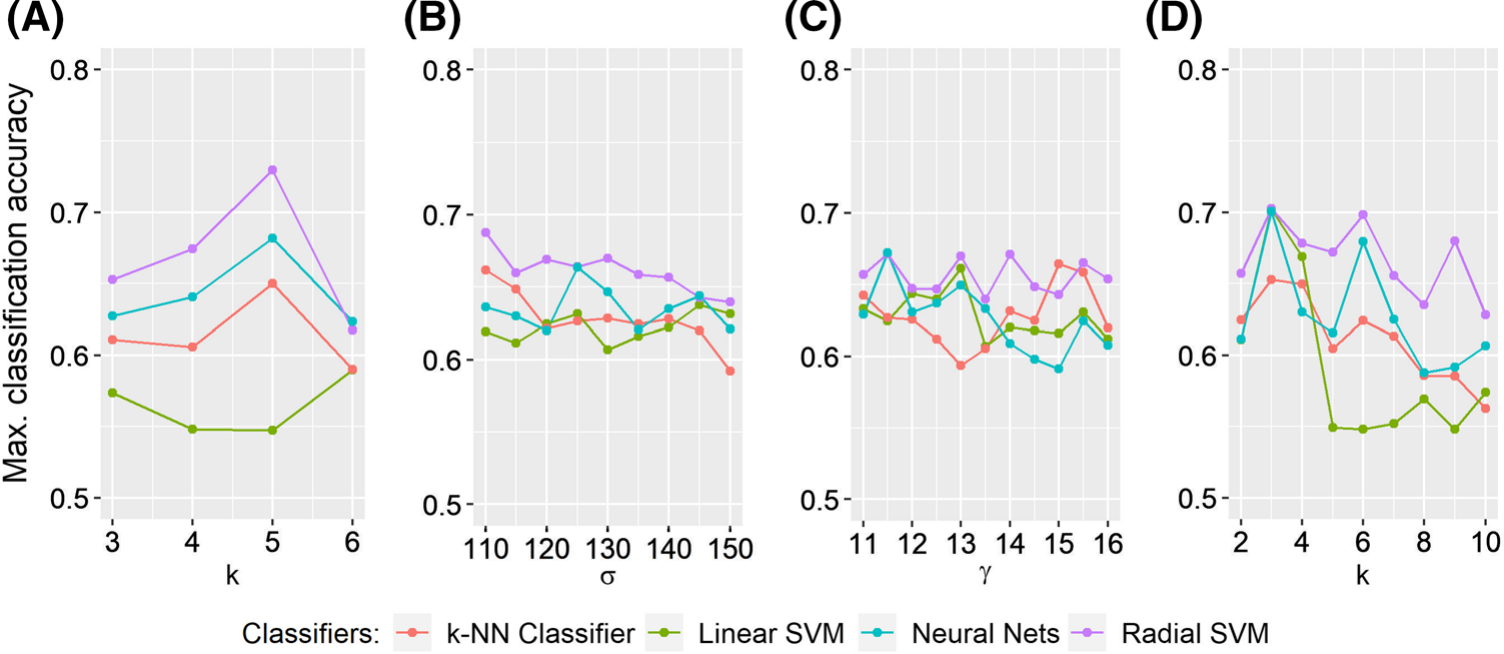
Classification performance of the parametric manifold learning techniques with respect to different parameter values. **A** ISOMAP (*k*), **B** diffusion maps ($\sigma$), **C** kPCA ($\gamma$), **D** LLE (*k*). The metric used is the the Euclidean distance $L_{2}$ (see “Construction of FCN based on the Euclidean distance” section)

Figure 8 depicts the accuracy of all methods across all thresholds and classifiers. Figure 9 shows the maximum classification accuracy of all parametric methods for different values of the parameters.

Here, the best classification accuracy was obtained with ISOMAP (72.9% using RSVM and 68 % PT ); ISOMAP slightly outperformed the Euclidean matrix (which yielded 72% using the k-nn classifier and 64% PT). The choice of *k* nearest neighbours affected the performance of ISOMAP as for any other *k* the accuracy was below 70% (Fig. 9A). The LSVM performed poorly for most manifold learning methods with the exception of the LLE (Fig. 8); LLE was more robust with respect to different thresholds (Fig. 8) with the maximum classification rates reaching 70% for the three out of the four classifiers used (only the k-NN classifier peaked at 65.3%); Its best performance was 70.3% using RSVM and 48% PT. LLE was again sensitive to the choice of *k* nearest neighbours as for larger numbers of *k*, the accuracy dropped for all classifiers (Fig. 9D). The kPCA was not robust against thresholding (Fig. 8) while different parameter values did not change much its performance (Fig. 9C). In terms of maximum classification accuracy kPCA performed worse than any other method used (Table 2), with a peak at 67.2% using ANN and 62% PT. Diffusion maps yielded a maximum classification of 68.8 % using RSVM and 66% PT; the performance was generally similar to the one of the Euclidean matrix, yet with a lower maximum classification rate; different values of the parameter $\sigma$ did not change a lot the classification rates for most classifiers (Fig. 9B). Finally, MDS yielded a 72.3% using RSVM and 54% PT. In terms of the maximum classification accuracy, MDS performed similarly to the Euclidean matrix and ISOMAP (Table 2). Finally, RSVM produced again the highest classification accuracy for most of the manifold learning algorithms.

**Fig. 10:**
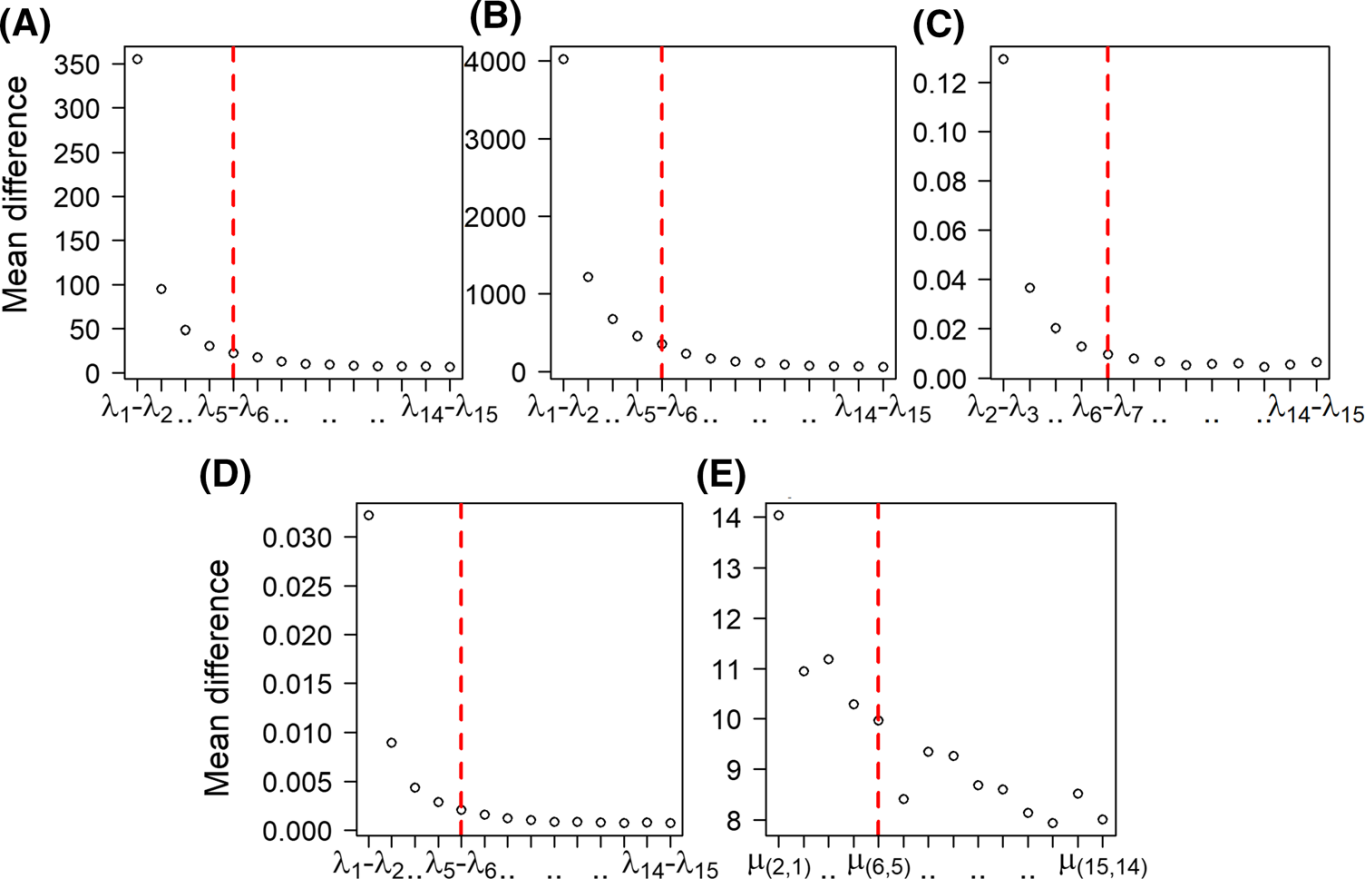
Mean differences of the 15 largest (smallest for the LLE) eigenvalues (see “Choice of the embedding dimension” section) for all manifold learning algorithms using the Euclidean distance $L_{2}$. **A** MDS (see  “Construction of FCN with MDS” section), **B** ISOMAP (see  “Construction of FCN using ISOMAP” section), **C** diffusion maps (see “Construction of FCN using diffusion maps” section), **D** kPCA (see “Construction of FCN using kernel principal component analysis” section), **E** LLE (see “Construction of FCN using locally linear embedding” section) using the optimal parameters. The red dashed vertical line marks the maximum number of dimensions considered (i.e. 5 dimensions, see “Choice of the embedding dimension” section). For the case of LLE, the function $\mu ({\mathbf {i,j}})=\frac{\mathbf {1}}{\lambda _{\mathbf {M-i}}-\lambda _{\mathbf {M-j}}}$ was used for visualization purposes as we are interested in the smallest eigenvalues (trivial eigenvalue $\lambda _{M} = 0$ is discarded). (Color figure online)

Finally, Fig. 10 depicts characteristic eigenspectrums for all manifold learning algorithms.

### Comparison between metrics

**Fig. 11:**
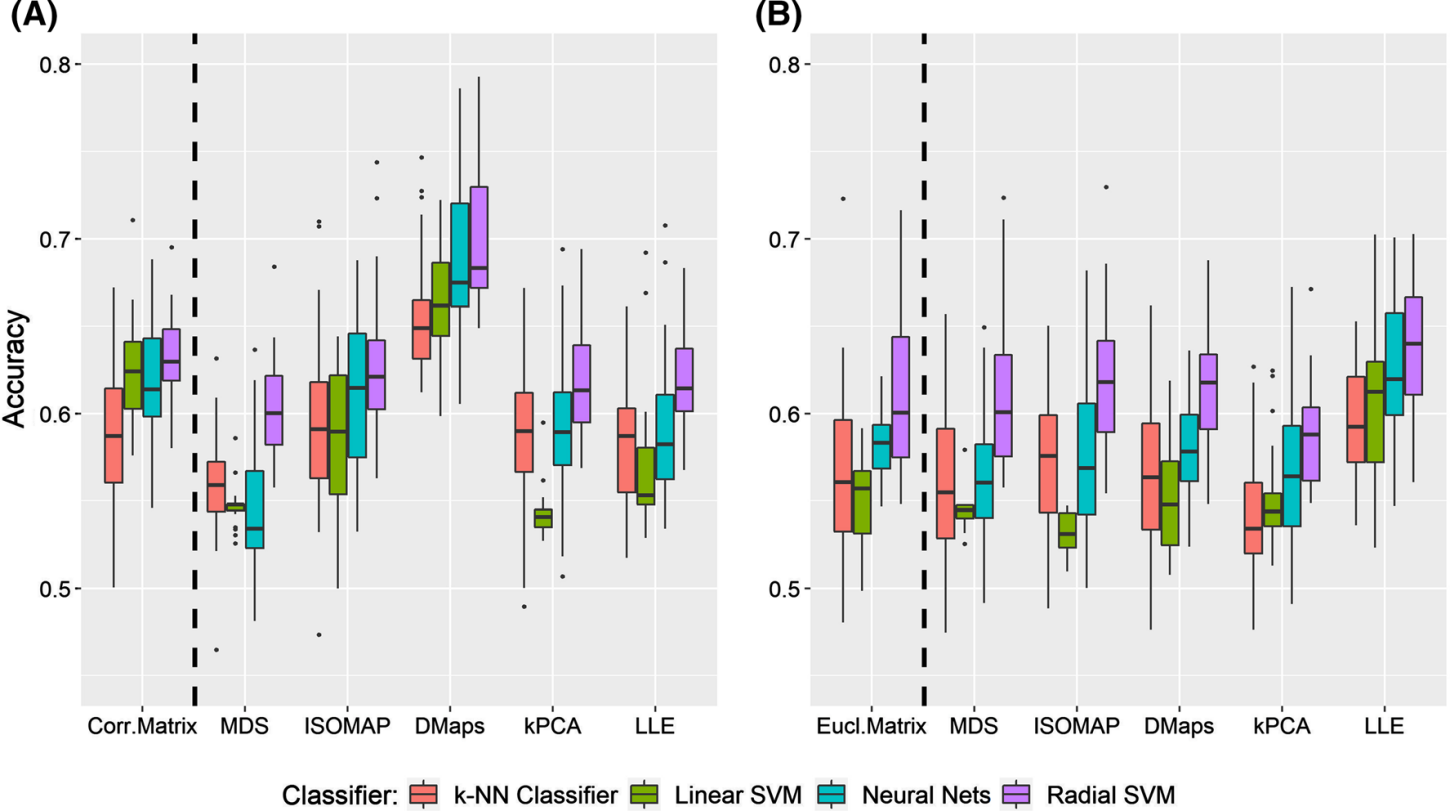
Boxplots of classification rates over all classifiers and thresholds, using the (**A**) cross correlation pseudo-distance $d_c$, (**B**) the Euclidean distance $L_{2}$. The labels at the bottom of each panel correspond to the method used for the construction of the FCN: the cross correlation matrix (Corr. Matrix), the Euclidean matrix (Eucl.Matrix), MDS, ISOMAP, diffusion maps(DMaps), kPCA, LLE. The black horizontal lines mark the median values of the distributions

Figure 11 illustrates the accuracy rates (as boxplots) for every method used based on the optimal parameters. The first panel (A) shows the accuracy rates when using the cross correlation metric, (B) shows the accuracy rates when using the Euclidean distance. At the extreme left of each panel the accuracy rates of the cross correlation matrix (Fig. 11A, Corr.Matrix) and the Euclidean matrix (Fig. 11B, Eucl.Matrix) are also shown. For each method, there are four boxplots of different colours, one for each classifier (i.e Linear SVM, Radial SVM, k-NN classifier and artificial Neural Nets). The black points denote an outlier of the distribution (here, the classification rates across all PTs) while the black horizontal lines mark the median value of the distribution.

#### Comparison between the cross correlation and the Euclidean matrix

The cross correlation matrix yielded in general better results compared to the Euclidean matrix judging by the overall performance [i.e the median classification rates depicted by the black horizontal lines (see Fig. 11A, B)].

#### Performance comparison of manifold learning methods

The general performance of MDS was relatively poor for both metrics. When using the cross correlation metric, the MDS was outperformed by any other method [the maximum classification accuracy for every classifier was lower compared to other methods (see Table 1)]. On the other hand, with the Euclidean metric, only the RSVM resulted in high classification rates, thus performing similarly and/or slightly better than the Euclidean matrix (see Table 2). ISOMAP performed similarly to the correlation matrix [when using the cross correlation metric (see Fig. 11A)] and the Euclidean matrix [when using the Euclidean distance (Fig. 11B)] but in both cases yielded better single maximum classification rates (74.4 % when using the correlation metric and 72.9% when using the Euclidean). An exception was the LSVM’s poor performance, especially when the Euclidean metric was used.

The diffusion maps with the cross correlation metric was superior to all other methods with respect to the overall performance, robustness against thresholding (Fig. 4) and maximum classification rate (see Table 1). The diffusion maps scored the best classification rate (79.3% using RSVM). However, this was not the case when using the Euclidean metric. The overall performance of diffusion maps with the Euclidean metric was similar to the one of the Euclidean matrix but with lower maximum classification rates for two (RSVM and k-NN) out of the four classifiers used.

The performance of kPCA was poor for both metrics. The linear classifier’s accuracy rates (in both cases) was in general under 60%. Only the RSVM classifier performed similarly and sometimes better than the cross correlation matrix when using the cross correlation metric. Using the Euclidean as a metric for kPCA all median classification rates for all classifiers was below 60% (Fig. 11B).

The LLE with the cross correlation metric performed relatively poor for three (knn classifier, LSVM and ANN) out of the four classifiers used. Only the RSVM classifier performed similarly to the cross correlation matrix. On the other hand, when using the Euclidean distance, the LLE performed better (Fig. 11B). Despite the fact that it did not produce the best maximum classification rate, LLE was more robust against different values of PT and reached 70 % classification accuracy for three out of the four classifiers used.

## Discussion

In this study, we constructed embedded FCN from rsfMRI data using linear and non-linear manifold learning techniques. Based on fundamental graph theoretic measures of the constructed FCN, we then used machine learning algorithms for classification purposes. We also compared the performance of two widely used metrics in neuroimaging, namely the cross correlation and the Euclidean distance. For our demonstrations, we used a publicly available dataset of resting-state fMRI recordings taken from healthy and patients with schizophrenia. This is the first study that performs such a systematic comparative analysis between various manifold learning algorithms, machine learning algorithms and metrics. To the best of our knowledge, it is also the first study that shows how the algorithm of diffusion maps can be exploited to construct FCN from rsfMRI data.

At this point we should note that our intention was not to try to obtain the best possible classification performance by “optimising” the pre-processing of the raw fMRI data and/or by trying to find the best set of graph-theoretic measures [other studies have already shed light towards this direction (Čukić et al. 2020; Xiang et al. 2020; Vergara et al. 2017)]. For example Čukić et al. (2020) showed that successful discrimination of depression from EEG could be attributed to proper feature extraction and not to a particular classification method. Thus, here we aimed at comparing mainly the manifold learning methods by factoring out the influence of a specific feature selection method; classification was based only on three key global graph-theoretic measures that are widely used in neuroscience (Stam and Reijneveld 2007; Bullmore and Sporns 2009), namely, the average path length, the global clustering coefficient and the median degree of the embedded binary networks. Indeed as also discussed in Bullmore and Sporns (2009) the general brain network can be described at a global scale by the shortest path length which is associated with the transfer of information, the clustering coefficient associated with robustness to random error, and the degree associated with the existence of hubs, and a modular community structure. Even though, we could consider a few more global graph measures, we decided not to do so, as other measures like the global efficiency (in essence, the inverse of average path length) or the diameter of a graph (the largest path length of a graph from one vertex to another) are highly correlated with one (or more) of the three basic measures stated above (e.g. the global efficiency is the inverse of characteristic path length and the diameter of a graph is likely to be higher as the average path length gets larger). Based on the above fundamental graph measures, our best reported accuracy obtained with diffusion maps and cross correlation was 79.3 % (evaluated with a 10 fold cross validation scheme, repeated 100 times). The conventional methodology was outperformed in both overall performance and with respect to the maximum classification accuracy as there was an 8.3% difference in classification accuracy in favor of the diffusion maps.

For the same benchmark fMRI dataset, Anderson and Cohen (2013) used ISOMAP for the construction of embedded FCN for the classification between heatlhy controls and schizophrenia patients. ROIs were acquired as here using single subject ICA and functional connectivity was accessed using the cross correlation distance. The analysis revealed differences in small-world properties among groups and 13 graph theoretic features led to a reported 65% accuracy rate. Xiang et al. (2020) reported a 93.1% accuracy (with sparse group Lasso and 78.6% with a Welch’s *t* test) testing more than 1000 graph-based features. An anatomical atlas was used for signal extraction and ROI selection, while an SVM-based classifier was used along with a “leave one out” scheme to evaluate its performance. In the same study, the authors compare their method with other novel approaches that have been previously proposed (Cheng et al. 2015; Huang et al. 2018) by applying them on the COBRE dataset. Cheng et al. (2015) calculated the betweenness centrality of nodes and used their ranks to classify patients with schizophrenia and healthy controls. For the COBRE dataset, this approach yielded a 74.4% classification accuracy. Finally, Huang et al. (2018) used a tree-guided group sparse learning method to extract key information in four frequency bands. Applied on the COBRE dataset, the classification performance peaked at 77.3%.

Our best reported accuracy (79.3% evaluated with tenfold cross validation repeated 100 times) is still higher than some of the previously proposed methods applied to the same dataset.

Some recent studies have suggested that the correlation matrices lie on a non-linear manifold (Venkatesh et al. 2020). Regarding our results, the diffusion maps algorithm (based on the diffusion distance) and the ISOMAP (based on the geodesic distance) managed to outperform the correlation matrix (that is most frequently used in constructing FCN from fMRI data) in terms of classification accuracy. However, other techniques such as the LLE (locally preserving the distance among neighbours), the gaussian kernel PCA (the non linear extension of PCA) and the MDS (which preserves the Euclidean distances on the embedded manifold) performed similarly or poorer compared to the cross correlation matrix. On the other hand, when using the Euclidean distance as a metric of functional connectivity none of the methods used in this study exhibited much higher results than the Euclidean matrix (which was slightly outperformed by ISOMAP and MDS). However, LLE provided a more robust classification pattern with most of the classifiers reaching a 70% accuracy. Though in general, there is no single best manifold learning method outperforming all the others for both metrics, our study showed that the diffusion maps result in higher classification accuracy when using the cross correlation distance (10.5% difference with respect to the Euclidean distance) outperforming the “conventional” method for constructing FCN from fMRI data.

However, this study does not come without limitations. For example, the method chosen for signal extraction is the single-subject ICA (Anderson and Cohen 2013). While this methodology holds the advantage of yielding subject-specific ICs (taking into account the within-subject variation), yet, due to this fact, we could not utilize local features in our graph theoretic analysis as the ICs (the nodes in the constructed graphs) were not the same across participants (even the number of ICs were different just like in Anderson and Cohen 2013). Thus, in order to factor out the influence of specific features, as discussed above, we used the three fundamental global theoretical measures to quantify differences among groups in the global topology of the network (Stam and Reijneveld 2007; Bullmore and Sporns 2009). An alternative method for the signal extraction could be the use of group-ICA. For example, a group-ICA analysis has been recently applied in a large fMRI dataset (151 healthy controls and 163 schizophrenia patients) for classification purposes (Salman et al. 2019); under this methodology the authors reached a maximum of 76.4% classification accuracy. While studies have shown that group-ICA can capture inter-subject spatial variability, it is not without limitations (Allen et al. 2012). For example group-ICA makes the assumption that each subject makes the same contribution to the observed “group” ICs, discarding random subject to subject variations. Thus, one cannot generalize the conclusions to the population (Friston et al. 1999). Hence, different approaches (such as the Independent Vector Analysis) have been suggested that seek for an optimal trade-off between group and individual representation, trying to preserve subject’s variability within a group (Michael et al. 2014).

## Data Availability

The COBRE dataset is publicly available at http://fcon_1000.projects.nitrc.org/indi/retro/cobre.html. For our analysis, we used the “R” software subroutines as described in “Materials and methods” section and “Appendix”.

## Appendix

For classification we used Linear Support Vector Machines (LSVM), Radial (Radial basis function kernel) Support Vector Machines (RSVM), one hidden layer Artificial Neural Networks (ANN) and k-NN classifier (k-NN). All classifiers were trained and evaluated via repeated tenfold cross validation scheme repeated 100 times. For the classification we used the three key graph theoretic measures as described in “Graph-theoretic measures” and “Materials and methods” sections. Training and classification were implemented using algorithms contained in package “caret” (Kuhn et al. 2008) publicly available in R free software environment (Team 2014).

### Support vector machines (SVM)

Support vector machines (SVM) aim at finding the optimal separating plane or hyperplane in the feature space among groups. In particular, for a set of points $({\mathbf {x}}_i,y_i)_{i=1,2\ldots N}$, where *N* is the number of subjects, ${\mathbf {x}}_i\in {\mathbf {R}}^d$ contains *d* attributes/features selected for subject *i* and $y_i \in (-1,1)$ the subject’s class (here, either healthy or patient), SVM attempts to find the optimal plane or hyperplane that divides the two classes by maximizing the margin of separation. Any hyperplane can be modelled as ${\mathbf {w}}\cdot {\mathbf {x}}_i+b=0$ where ${\mathbf {w}}$ represent the weights of features ${\mathbf {x}}_i$. Parallel hyperplanes can be described as ${\mathbf {w}}\cdot {\mathbf {x}}_i+b \ge 1$ if $y_i=1$ and ${\mathbf {w}}\cdot {\mathbf {x}}_i+b \le -1$ if $y_i=-1$. The optimization problem then aims at maximizing the margin between hyperplanes $\frac{2}{\Vert {\mathbf {w}}\Vert }$ such that for every $(y_i)_{i=1,2\ldots N}, \, y_i\cdot ({\mathbf {w}}\cdot {\mathbf {x}}_i+b) \ge 1$.

One can take advantage of a regularization parameter *C* indicating the penalty of error $z_i$ that gives a trade-off between misclassifications and the width of the separating margin. This leads to the final optimization problem, which minimizes $\frac{\Vert {\mathbf {w}}\Vert ^2}{2}+ C\cdot \sum _{i}z_i$ subject to $y_i\cdot ({\mathbf {w}} \cdot {\mathbf {x}}_i+b) \ge 1-z_i$,   $i=1,2\ldots N$.

Based on the idea that the data maybe better separable in a higher dimensional space, SVM may utilize a kernel function to map ${\mathbf {x}}_i\in {\mathbf {R}}^d$ to $\phi ({\mathbf {x}}_i)\in {\mathbf {R}}^D$, $D>d$. In our study, besides standard linear SVM (LSVM), we also used radial SVM (RSVM) making use of the radial basis functions kernel given by $K({\mathbf {x}}_i,{\mathbf {x}}_j)=exp\left( -\frac{\Vert {\mathbf {x}}_i-{\mathbf {x}}_j\Vert ^{2}}{2\cdot \gamma ^2}\right)$, where $\gamma$ is the kernel’s scale parameter.

### k-Nearest neighbours classifier(k-NN)

k-Nearest neighbours algorithm is one of the simplest classification/machine learning algorithms. Given $({\mathbf {x}}_i,y_i)_ {i=1,2\ldots N}$, where *N* is the number of subjects, ${\mathbf {x}}_i\in {\mathbf {R}}^d$ contains *d* attributes/features selected for subject *i* and $y_i$ the subject’s class (here, either healthy or patient), k-NN utilizes Euclidean distance in the feature space to perform a voting system among *k* closest neighbours. In this manner, each point is classified as “control”, if the number of “control” neighbours is greater than the number of “patient” neighbours and inversely. The number *k* of closest neighbours is a parameter of choice that plays a crucial role in method’s performance. In this study, it is important to note that we chose odd values of *k* (i.e how many neighbours we take into consideration) in order not to have to break possible ties in the voting system among neighbours

### Artificial neural networks (ANN)

In this study, we also used feed-forward artificial neural networks (ANN) consisting of one hidden layer. The input units were three as the number of the features considered for classification. We have chosen one hidden layer consisting from 1 to 5 neurons along with a bias term. The activation function used for all neurons was the logistic transfer function (Ripley 2007). The output was one node (reflecting simple binary classification control/patient). The training procedure of the model was done via back-propagation (Hecht-Nielsen 1992) using a tenfold cross validation scheme. Finally a weight decay parameter *a* (regularization parameter) was used to prevent over-fitting and improve generalization (Krogh and Hertz 1992) of the final model. For the implementation of the ANN we used the “nnet” software package (Ripley and Venables 2011) publicly available in R free software environment (Team 2014).

### Parameters tested for each classifier

We tuned the parameters of the algorithms via grid search.

For the SVM:

$$\begin{aligned} C&= (0.1,0.25,0.5,0.75,1,2.5,5,7.5,10,\\&25,50,75,100,250,500,750,1000)\\ \gamma&= (0.001,0.01,0.1,0.25,0.5,0.75,1,2.5,5,\\&7.5,10,25,50,75,100,250,500,750,1000). \end{aligned}$$

For the k-NN classifier: $\kappa = (1,3,5,7,9)$.

For the ANN: number of neurons in the hidden layer $p = (1,2,3,4,5)$, decay level $a= (0.0001,0.001,0.01,0.025,0.05,0.075,0.1)$.
